# Molecular basis of *FAAH-OUT*-associated human pain insensitivity

**DOI:** 10.1093/brain/awad098

**Published:** 2023-05-24

**Authors:** Hajar Mikaeili, Abdella M Habib, Charlix Wai-Lok Yeung, Sonia Santana-Varela, Ana P Luiz, Kseniia Panteleeva, Sana Zuberi, Alkyoni Athanasiou-Fragkouli, Henry Houlden, John N Wood, Andrei L Okorokov, James J Cox

**Affiliations:** Molecular Nociception Group, Wolfson Institute for Biomedical Research, University College London, London WC1E 6BT, UK; College of Medicine, QU Health, Qatar University, Doha, Qatar; Molecular Nociception Group, Wolfson Institute for Biomedical Research, University College London, London WC1E 6BT, UK; Molecular Nociception Group, Wolfson Institute for Biomedical Research, University College London, London WC1E 6BT, UK; Molecular Nociception Group, Wolfson Institute for Biomedical Research, University College London, London WC1E 6BT, UK; Molecular Nociception Group, Wolfson Institute for Biomedical Research, University College London, London WC1E 6BT, UK; Molecular Nociception Group, Wolfson Institute for Biomedical Research, University College London, London WC1E 6BT, UK; Department of Molecular Neuroscience, Institute of Neurology, University College London, London WC1N 3BG, UK; Department of Molecular Neuroscience, Institute of Neurology, University College London, London WC1N 3BG, UK; Molecular Nociception Group, Wolfson Institute for Biomedical Research, University College London, London WC1E 6BT, UK; Molecular Nociception Group, Wolfson Institute for Biomedical Research, University College London, London WC1E 6BT, UK; Molecular Nociception Group, Wolfson Institute for Biomedical Research, University College London, London WC1E 6BT, UK

**Keywords:** endocannabinoid system, pain, pseudogene, regulatory RNA, anandamide

## Abstract

Chronic pain affects millions of people worldwide and new treatments are needed urgently. One way to identify novel analgesic strategies is to understand the biological dysfunctions that lead to human inherited pain insensitivity disorders. Here we report how the recently discovered brain and dorsal root ganglia-expressed *FAAH-OUT* long non-coding RNA (lncRNA) gene, which was found from studying a pain-insensitive patient with reduced anxiety and fast wound healing, regulates the adjacent key endocannabinoid system gene *FAAH*, which encodes the anandamide-degrading fatty acid amide hydrolase enzyme.

We demonstrate that the disruption in *FAAH-OUT* lncRNA transcription leads to DNMT1-dependent DNA methylation within the *FAAH* promoter. In addition, *FAAH-OUT* contains a conserved regulatory element, FAAH-AMP, that acts as an enhancer for *FAAH* expression.

Furthermore, using transcriptomic analyses in patient-derived cells we have uncovered a network of genes that are dysregulated from disruption of the *FAAH-FAAH-OUT* axis, thus providing a coherent mechanistic basis to understand the human phenotype observed.

Given that *FAAH* is a potential target for the treatment of pain, anxiety, depression and other neurological disorders, this new understanding of the regulatory role of the *FAAH-OUT* gene provides a platform for the development of future gene and small molecule therapies.

## Teaser

Mikaeili *et al*. identify the molecular basis of *FAAH-OUT*-associated pain insensitivity, in which painlessness is accompanied by reduced anxiety and accelerated wound healing. The *FAAH-OUT* long non-coding RNA gene regulates *FAAH* expression via two transcription-dependent mechanisms, providing a platform for future gene therapy.

## Introduction

Millions of people worldwide are living in chronic pain.^1^ To compound the problem, the over-prescription of opioid-based drugs to treat pain has contributed to an opioid epidemic that is causing significant morbidity and mortality, particularly in the USA.^2^ In the UK, chronic pain affects up to 50% of adults and about 12% of those have moderate-to-severe disabling pain.^3^ This has been further aggravated by the Covid-19 pandemic with up to 2 million people in the UK experiencing ‘long Covid’ symptoms that include pain, depression and anxiety.^4^ Poorly treated chronic pain therefore makes life intolerable for extreme numbers of people and new pain-killing medications are hence urgently needed.

The endogenous cannabinoid [endocannabinoid, (eCB system or eCBS)] affects a diverse array of key physiological functions including anxiety and stress responses, pain modulation, learning and memory, wound healing and development.^5^ It comprises the CB1 and CB2 G protein-coupled cannabinoid receptors, eCB lipid ligands [anandamide (AEA) and 2-arachidonoylglycerol (2-AG)] and their synthesizing [e.g. *N*-acyl phosphatidylethanolamine phospholipase D (NAPE-PLD)] and metabolizing [fatty acid amide hydrolase (FAAH) and monoacylglycerol lipase (MAGL)] enzymes.^6^ The expanded eCBS includes oleoylethanolamide (OEA) and palmitoylethanolamide (PEA) lipid mediators, their receptors (e.g. TRPV1 and PPARα) and metabolic enzymes. Components of the eCBS are potential therapeutic targets for a wide range of neurological conditions including chronic pain, anxiety and depression, as well as neurodegenerative conditions such as Alzheimer’s and Parkinson’s diseases.^5^

A key target in the eCBS is fatty acid amide hydrolase, an important catabolic enzyme that degrades AEA, OEA, PEA and other lipids such as *N*-acyltaurines (NATs).^7-9^ FAAH is particularly enriched in the liver, brain and also expressed within trigeminal and dorsal root ganglia (DRG).^10-13^ Within the brain, *FAAH* is found in regions that are significant for nociceptive transmission and modulation including the thalamus, periaqueductal grey (PAG) and amygdala. *Faah* is expressed in approximately a third of rat DRG neurons, with ∼70% of these being TRPV1-positive.^12^ Following sciatic nerve axotomy, expression of *Faah* is also induced in large diameter DRG neurons.^12^ Over the past 20 years many FAAH-inhibiting drugs have been developed, although none has yet successfully reached the clinic after human trials.^14-17^ Unfortunately, a lethal toxic cerebral syndrome was precipitated by a recently trialled FAAH inhibitor (BIA 10-2474) that was later shown to be related to off-target effects.^18,19^

A powerful way to identify novel human-validated analgesic drug targets is to study rare individuals with intact damage-sensing neurons that present with a congenital pain insensitive phenotype.^20^ Recently we reported a new pain insensitivity disorder after studying a female patient (Patient PFS) who, in addition to being pain insensitive, also presented with additional clinical symptoms including a happy, non-anxious disposition, fast wound-healing, reduced stress and fear symptoms, mild memory deficits and significant postoperative nausea and vomiting induced by morphine.^21^ This phenotype was consistent with enhanced eCB signalling and genetic analyses showed two distinct mutations: (i) a microdeletion in a DRG and brain-expressed long non-coding RNA (lncRNA)-expressing pseudogene, *FAAH-OUT*, which is adjacent to the *FAAH* gene on human chromosome 1; and (ii) a common functional single-nucleotide polymorphism in *FAAH*, conferring reduced *FAAH* expression and activity.^22,23^ These mutations result in enhanced levels of anandamide and other bioactive lipids, that are normally degraded by FAAH.^21^

Despite *FAAH* being a heavily researched gene, the *FAAH-OUT* gene locus and how it regulates *FAAH* expression have been overlooked. Here we set up to elucidate how the ∼8 kb microdeletion that is distinct from and begins ∼5 kb downstream of the 3’ end of the currently annotated footprint of the *FAAH* gene disrupts its function. Potential key mechanisms we considered included (i) the microdeleted genomic sequence contains important regulatory elements needed for normal *FAAH* expression (e.g. an enhancer); and (ii) the *FAAH-OUT* lncRNA transcript has an epigenetic/transcriptional role in regulating *FAAH* expression.

Here we show by gene editing in human cells that the ∼8 kb region that is deleted in Patient PFS results in reduced expression of *FAAH*. We also demonstrate that the *FAAH-OUT* lncRNA is enriched in nuclei and its transcription positively correlates with expression of *FAAH*, bearing all the trademarks of a positive regulator. The reduction in *FAAH-OUT* transcription leads to enhanced DNA Methyltransferase 1 (DNMT1)-dependent DNA methylation of the CpG island within the *FAAH* gene promoter, resulting in transcriptional shutdown of *FAAH. FAAH-OUT* therefore appears to regulate *FAAH* expression via preventing DNMT1-dependent DNA methylation of the *FAAH* promoter, thus maintaining its transcriptional potential.

Furthermore, we show that the *FAAH-OUT* microdeletion region contains a conserved regulatory element within the first intron of *FAAH-OUT*, FAAH-AMP, that behaves as an active enhancer regulating *FAAH* expression. Editing or silencing the *FAAH-OUT* promoter region or the short evolutionarily conserved FAAH-AMP element leads to reduced *FAAH* mRNA in human cells.

Finally, to narrow in on the key functional targets downstream of the *FAAH − FAAH-OUT* axis, we used microarray analysis of Patient PFS-derived fibroblasts to uncover a network of key molecular pathways and genes that become dysregulated as a result of activity disabling mutations in the *FAAH* and *FAAH-OUT* genes.

## Materials and methods

### Transient transfection of CRISPR/Cas9 plasmids into HEK293 and CAD cells

Human embryonic kidney 293 cells (HEK293, ECACC) were cultured in Dulbecco’s modified Eagle medium (DMEM, Thermo Fisher) with 10% foetal bovine serum (FBS, Hyclone). Mouse catecholaminergic neuronal tumour cells (CAD, ECACC) were cultured in DMEM:HAMS F12 (1:1) with 2% glutamine and 8% FBS. Lipofectamine 3000 (Invitrogen) was used for DNA transfections (see plasmids in Supplementary Table 1) according to the manufacturer’s procedures at DNA-Lipofectamine ratio 1:1 using 70% confluent HEK293/CAD cells. After 24 h of incubation at 37 °C, media was removed and the transfection steps were repeated. The cells were incubated at 37°C in a 5% CO_2_ incubator with 92–95% humidity for another 24 h.

To extract total RNA from cultured cells, medium was first aspirated off and cells were rinsed with ice cold phosphate-buffered saline (PBS). TRIzol^®^ (Invitrogen, 1 ml) was added directly to the cells and was incubated for 5 min at room temperature. Cell lysate was passed through a pipette up and down several times. RNA was extracted using PureLink™ RNA Micro Scale Kit (Invitrogen) according to the manufacturer’s procedures. Genomic DNA was isolated using the DNeasy Blood and Tissue kit (Qiagen) and used as template to confirm gene editing (Supplementary Table 2).

### Generation of stable cell lines

HEK293 cells were transfected using Lipofectamine 3000 with SaCas9-IRES-AcGFP1 plasmids containing guide pairs HMa or HMb (Supplementary Table 1) or a no guide control. For fluorescence-activated cell sorting (FACS), cells were washed with PBS and detached using trypsin. The cell pellets were washed twice with PBS and resuspended in ice cold PBS, 5 mM EDTA, 25 mM HEPES buffer and 1% FBS. The top 3% green fluorescent protein (GFP)-positive cells were sorted into 96-well plates (one cell per well) and cultured for 3 weeks. Genomic DNA was isolated using the DNeasy Blood and Tissue kit (Qiagen) and screened for the intended deletion by PCR, with primers flanking and internal to the microdeletion (Supplementary Table 2).

### TaqMan real-time PCR

Reverse transcription was performed using oligo d(T) and Superscript III first-strand synthesis system (Invitrogen) according to the manufacturer’s conditions. TaqMan real-time PCR was carried out using the following probes for human genes: *FAAH* (Hs01038660_m1), *FAAH-OUT* (Hs04275438_g1), *BDNF* (Hs03805848_m1), *ACKR3* (Hs00604567_m1), *WNT5B* (Hs01086864_m1), *GABBR2* (Hs01554996_m1), *DKK1* (Hs00183740_m1), *SFRP2* (Hs00293258_m1), *SERPINF1* (Hs01106937_m1) and *ACTB* (Hs01060665_g1). Mouse TaqMan probes used were: *Faah* (Mm01191801_m1) and *Actb* (Mm01205647_g1). The expression level of target genes was normalized to the housekeeping Actin gene mRNA. Relative gene expression [relative quantities (RQ) value] was determined using the 2^−ΔΔCt^ equation in which control unaffected individuals or empty vector cDNA samples were designated as the calibrator. All RT–PCR data are expressed as mean ± standard error of the mean (SEM) with significance indicated by *P ≤ 0.05, **P ≤ 0.01 and ***P ≤ 0.001 (two-tailed Student’s *t*-test).

### Chromatin immunoprecipitation

Chromatin immunoprecipitation (ChIP) assays were performed according to the manufacturer’s protocol using the Chromatrap Enzymatic ChIP-seq kit. Immunoprecipitations were performed overnight at 4°C using antibodies against H3K27ac (Abcam 4729), H3K4me1 (Abcam 8895), H3K4me3 (Abcam 8580), H3K9me3 (Abcam 8898), H3K27me3 (Active Motif 39157), DNMT1 (Active Motif 39204) and DNMT3A (Active Motif 39206). Rabbit IgG were used as control for ChIP and primers within a gene desert on chromosome 16 were used as a negative control for qPCR.

All ChIP experiments were performed in triplicates using two independent chromatin preparations. The immunoprecipitated DNA and the input DNA were analysed by real-time PCR using the ΔΔCt method and the primers are listed in Supplementary Table 3.

### EpiTect methyl II PCR assay

EpiTect methyl II PCR primer assay (Qiagen) was performed according to the manufacturer’s protocol. Briefly, 250 ng of genomic DNA was used to set up the four independent restriction enzyme digests: (i) methylation-sensitive; (ii) methylation-dependent; (iii) methylation-sensitive and methylation-dependent double digest; or (iv) mock digest. Q-PCR was performed as per the manufacturer’s protocol, using commercially available primers for human *FAAH* (CpG Island 100530) (EPHS100530-1A, Qiagen). Methylation-sensitive (EPHS115450-1A) and methylation-dependent (EPHS115451-1A) digest control assays were performed to test the cutting efficiency of the restriction enzymes. Samples were analysed as recommended by the manufacturer (Qiagen).

### Fibroblast cell lines

Ethical approval was granted by University College London REC and written informed consent was provided by Patient PFS and four gender-matched healthy control subjects. A punch skin biopsy (3–6 mm) was taken from the outer upper arm of each individual and primary cultures of dermal fibroblasts were passaged in DMEM (Thermo Fisher) supplemented with 10% FBS (Hyclone) and 1% penicillin/streptomycin (Thermo Fisher).

### Microarrays

Total RNA was isolated from the primary fibroblast cultures derived from Patient PFS and four healthy unrelated gender-matched control subjects (three homozygous for wild-type C allele at rs324420 and one heterozygous C/A) using the PureLink RNA Micro Kit (Invitrogen) and run by Eurofins on the human Clariom D transcriptomic array (Thermo Fisher) using the GeneChip WT Plus labelling kit reagent. Expression data were RMA normalized and analysed using the Transcriptome Analysis Console (TAC) software (Thermo Fisher) and Ingenuity Pathway Analysis (Qiagen). Microarray data have been deposited at Gene Expression Omnibus Array Express with reference number E-MTAB-11809.

### RNAscope *in situ* hybridization

DRG paraffin sections (HP-240, 5 µm thick), human brain cerebral cortex frozen sections (HF-210, 7–10 µm thick), human cerebellum frozen sections (HF-202, 7–10 µm thick) and human prostate frozen sections (HF-408, 7–10 µm thick) were obtained commercially from Zyagen (www.zyagen.com) via AMS Biotechnology (https://www.amsbio.com) (Fig. 2 and Supplementary Figs 1 and 2).

For the mouse DRG sections (Supplementary Fig. 3), adult C57BL/6 mice were deeply anaesthetized with pentobarbital (i.p.) and transcardially perfused with heparinized saline (0.9% NaCl) followed by 25 ml of cold 4% paraformaldehyde in PBS (pH 7.4). DRGs were extracted from the lumbar area and post-fixed with the same fixative solution for 2 h at 4 °C before being embedded in cryopreservative solution (30% sucrose) overnight at 4°C. Tissue samples were then placed in OCT blocks for posterior sectioning by cryostat. Sections (11 μm thick) were mounted onto Superfrost Plus (Fisher Scientific) slides, allowed to freeze-dry overnight at −80 °C, for an immediate use, or were stored at −80 °C in air-tight containers for no longer than a month for subsequent experiments.

*In situ* hybridization (ISH) was performed using the RNAscope assay (Advanced Cell Diagnostics) following the protocol for fresh-frozen samples for human cerebral cortex, human cerebellum and human prostate tissue samples, and mouse DRG samples using Multiplex Fluorescence Kit v2. Human DRG paraffin sections were treated according to the ACD’s FFPE-fixed samples protocol.

Probes included *hsNEFH* (#448141-c4), *hsCNR1* (#591521-c4), *hsFAAH* (#534291-c2) and *hsFAAH-OUT* (#534301-c3). RNA localization was detected with either AF488 or Opal 520 (green), Opal 570 (red) and Opal 650 (far-red) fluorochrome dyes (Perkin Elmer) compared to DAPI staining (nuclei) or TS-coumarin (TS405, Perkin Elmer) used for *NEFH* or *CNR1*. ISH slides were mounted using Prolong Gold (ThermoFisher Scientific #P36930). Mouse RNAscope probes included *mmFaah* (#453391) and *mmNefh* (#443671-c4).

Fluorescence was detected using Zeiss LSM 880 Airyscan microscope. Images were taken at 10 × and 20 × magnifications with 4 × averaging. Tiles were stitched when more than one was used to image the area, Airyscan processed and exported as 16-bit uncompressed tiff files for further basic editing in Adobe Lightroom v6 (Adobe) on a colour calibrated iMac (X-Rite) retina monitor. Final images were exported as jpeg files with 7200 pix on longest side at 300 ppi.

### Statistical analysis

Data were analysed using GraphPad Prism 9 (GraphPad Software, Inc), and results presented as mean ± SEM with *n* referring to the number of samples tested per group, as indicated in the figure legends.

### Data availability

Microarray data have been deposited at Gene Expression Omnibus Array Express with reference number E-MTAB-11809. All data are available in the main text or the Supplementary material.

## Results

### Gene editing mimicking the *FAAH-OUT* microdeletion reduces *FAAH* expression

**Figure 1:**
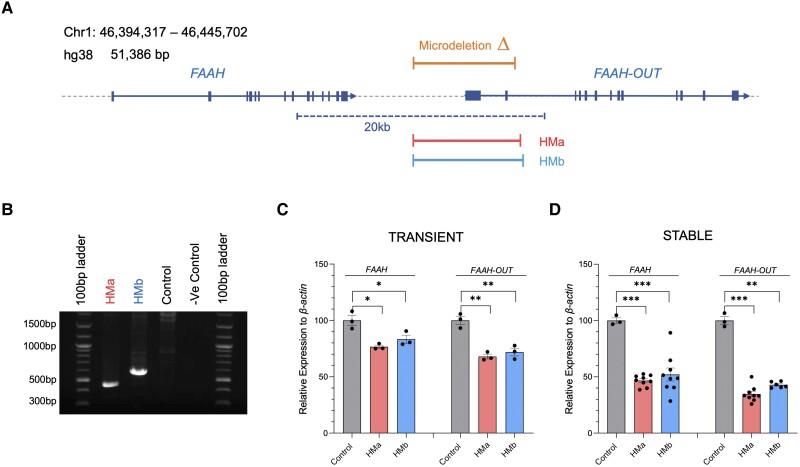
**Gene editing mimicking the *FAAH-OUT* microdeletion reduces FAAH expression.** (**A**) *FAAH* and *FAAH-OUT* genomic region. Map showing human chromosome 1 (46,394,317–46,445,702; build hg38). *FAAH* and *FAAH-OUT* genes are shown with exons denoted by blue boxes; the direction of transcription shown by arrows. The *FAAH-OUT* gene is composed of 13 exons and the microdeletion contains the first two exons and putative promoter region. The ∼8 kb microdeletion identified in Patient PFS is shown by the orange bar. Gene editing guide pairs HMa (in red) and HMb (in blue) flank the microdeleted region. (**B**) CRISPR/Cas9-induced microdeletion in HEK293 cells. Gel electrophoresis of PCR products produced with primers that flank the gene editing HMa and HMb guide pairs; template genomic DNA isolated from HEK293 transiently transfected (48 h) with the SaCas9 plasmids. Gene editing is detected by a ∼463 bp fragment amplified from HMa edited cells and a ∼598 bp fragment from HMb edited cells. No band is observed from empty vector (control) transfected cells indicating no editing at this locus. The large size of the unedited allele is beyond the capability of the DNA polymerase. (**C** and **D**) The microdeletion in *FAAH-OUT* leads to a significant reduction in both *FAAH-OUT* and *FAAH* expression. RT-qPCR analysis of both *FAAH-OUT* and *FAAH* mRNA levels following transient (**C**) and stable (**D**) transfections with HMa or HMb SaCas9 plasmids show significant reduction in both *FAAH-OUT* and *FAAH* expression levels. The normalized expression value of empty vector with SaCas9 but no guide RNA was set to 100, and all other gene expression data were compared to that sample. Data-points are denoted by dots, bars show the ± SEM, and data analysed by a Student’s *t*-test, *P ≤ 0.05, **P ≤ 0.01 and ***P ≤ 0.001.

Patient PFS carries a 8131 bp heterozygous microdeletion on chromosome 1 (hg38, chr1:46,418,743–46,426,873) that begins ∼4.9 kb downstream of the end of the *FAAH* gene (Fig. 1A).^21^ The microdeletion contains the first two exons and putative promoter region of *FAAH-OUT (FAAHP1*; GenBank KU950306), a novel 13-exon lncRNA that is classed as a *FAAH* pseudogene and which has a similar tissue expression profile to *FAAH.*^21^

In order to elucidate the role of the *FAAH-OUT* microdeletion on *FAAH* gene expression, we used the CRISPR/Cas9 system to edit human embryonic kidney cell lines (HEK293) to mimic the patient’s microdeletion. HEK293 cells were transiently transfected for 48 h with an SaCas9 plasmid bearing a guide pair (HMa or HMb, Supplementary material and Supplementary Table 1) that targets sequences flanking the microdeletion, with each showing the expected genomic deletion (Fig. 1B). Next, total RNA was isolated from the transiently transfected HEK293 cells (thus a mixture of transfected and untransfected cells) and reverse transcribed into cDNA. Quantitative real-time PCR showed a significant reduction in both *FAAH-OUT* and *FAAH* mRNAs for cells transfected with each set of guide pairs (HMa and HMb) that flank the microdeletion, highlighting that *FAAH* expression is affected by the induced downstream deletion (Fig. 1C).

We repeated the gene editing experiments making stable HEK293 cell lines heterozygous for the *FAAH-OUT* microdeletion by transfecting SaCas9-IRES-AcGFP1 DNA plasmids carrying the HMa or HMb guide pairs. GFP-positive cells were FAC sorted to single cells to generate monoclonal lineages, and the site-specific microdeletion was confirmed by genomic DNA PCR. RT-qPCR data on *FAAH* and *FAAH-OUT* expression levels in these stable cell lines heterozygous for the *FAAH-OUT* microdeletion confirmed that *FAAH* expression is affected by the induced downstream deletion with a ∼50% reduction in *FAAH* transcript detected (Fig. 1D).

### *FAAH-OUT* transcript is enriched in the nucleus

The *FAAH-OUT* transcript is classified as a lncRNA; it lacks a conserved protein-coding sequence, is more than 200 bp in length and is post-transcriptionally capped and polyadenylated.^24^ Studying its subcellular localization is a necessary step toward understanding the nature and mechanisms of its molecular functions.

We have shown previously that *FAAH-OUT* is expressed in a wide range of human tissues, including brain and DRG.^21^ Here we assessed the intracellular distribution of *FAAH* and *FAAH-OUT* transcripts using a highly sensitive fluorescence *in situ* hybridization (FISH) technology—RNAscope assay and confocal microscopy. To ensure the specific detection of *FAAH* and *FAAH-OUT* transcripts, we used probes that target different regions of each transcript.

**Figure 2:**
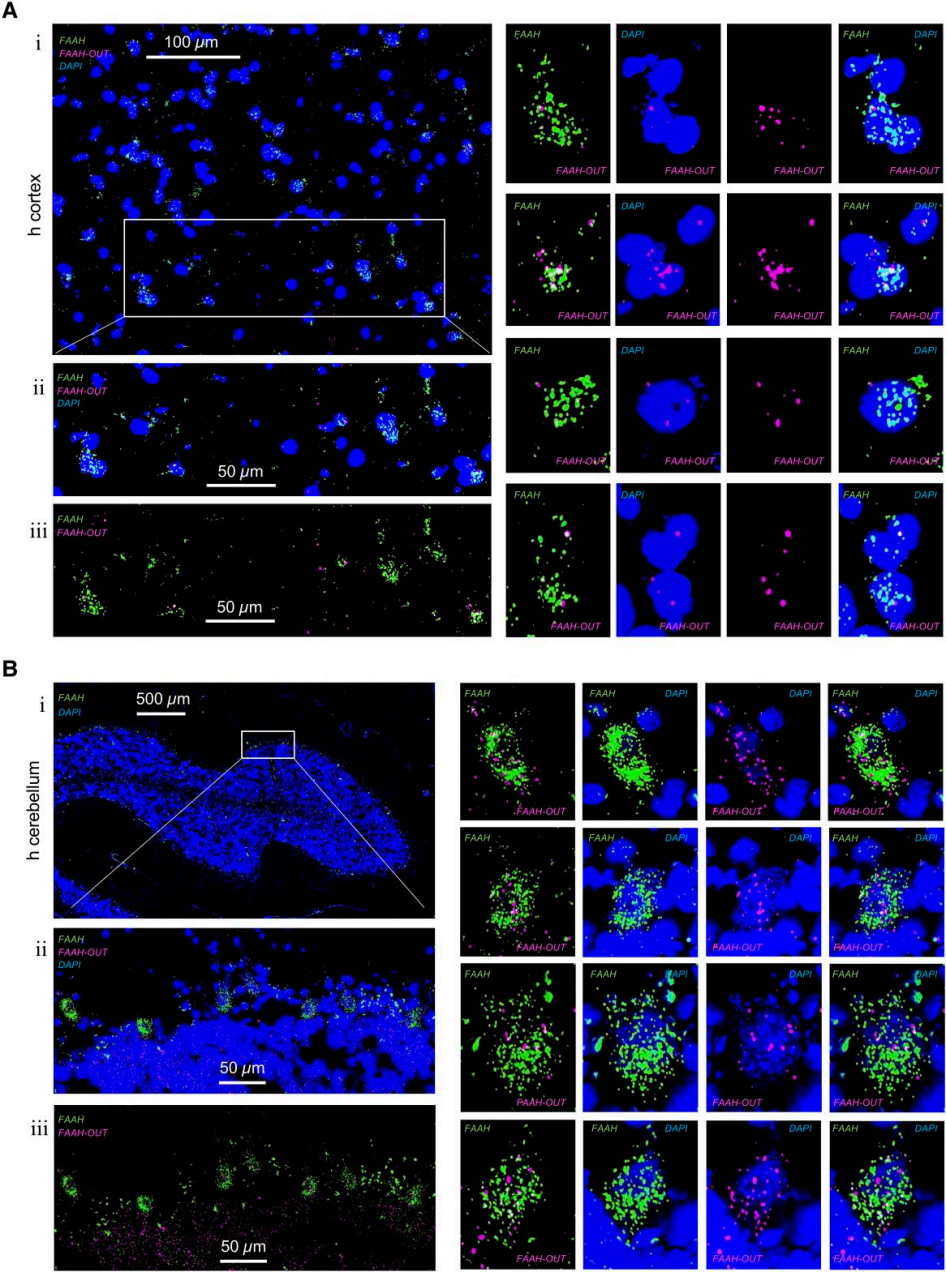
***FAAH* and *FAAH-OUT* RNA expression levels and localization in human brain tissue cells.** (**A**) *FAAH* and *FAAH-OUT* RNA expression levels and localization in human cerebral cortex cells. Cuts of fresh frozen cerebral cortex sections (7–10 μM thick) were analysed by RNAscope assay. Localization of *FAAH* mRNA (green, AF488) was compared to *FAAH-OUT* lncRNA (magenta, Opal650) localization and DAPI staining indicating nuclei positions (blue). Scale bars are in white. A representative area (**i**) and enlarged sub-area indicated by white box show colocalization of green (*FAAH*) and magenta signal (*FAAH-OUT*) to the same cells (**ii** and **iii**). Panels with zoomed-in individual cells expressing both *FAAH* mRNA (in green) and *FAAH-OUT lncRNA* (in magenta) are shown on the *right*. (**B**) *FAAH* and *FAAH-OUT* RNA expression levels and localization in human cerebellum cells. Fresh frozen cortex sections (7–10 μM thick cuts) were analysed by RNAscope assay. Localization of *FAAH* mRNA (green, AF488) was compared to *FAAH-OUT* lncRNA (magenta, Opal650) localization and DAPI staining indicating nuclei positions (blue). A representative area (**i**) and enlarged sub-area indicated by white box show colocalization of green signal (*FAAH*) and magenta signal (*FAAH-OUT*) to the same large neuronal cells (Purkinje cells) located at the outer edge of cerebellar folium (**ii** and **iii**). Panels with zoomed-in individual cells expressing both *FAAH* mRNA (in green) and *FAAH-OUT lncRNA* (in magenta) are shown on the *right* and demonstrate that *FAAH* mRNA is predominantly cytoplasmic whereas *FAAH-OUT* lncRNA is enriched in the nucleus. Scale bars are in white.

The simultaneous visualization of *FAAH* and *FAAH-OUT* transcripts in fresh-frozen (FF) and formalin-fixed paraffin-embedded (FFPE) human tissue samples (cortex, cerebellum, prostate and DRG) provided direct evidence that *FAAH* mRNA and *FAAH-OUT* lncRNA were expressed within the same cells and predominantly localized in the cytoplasm and nucleus, respectively (Fig. 2A and B and Supplementary Figs 1 and 2). *FAAH* mRNA levels were consistently highest in *NEFH*-positive neurons in human cortex and mouse DRG (Supplementary Figs 1B and 3). Consistent with the FISH data, subcellular fractionation of HEK293 cultures followed by RT-qPCR analysis demonstrated *FAAH-OUT lncRNA* is enriched in the nucleus when compared to the *FAAH* coding mRNA, which was enriched in the cytoplasmic fraction of cells (Supplementary Fig. 1D).

### Modulation of *FAAH-OUT* transcription affects *FAAH* expression

To explore what effect *FAAH-OUT* transcription has on *FAAH* expression levels we used CRISPR/Cas9 to either (i) delete the putative *FAAH-OUT* promoter region; or (ii) epigenetically silence the promoter using CRISPR interference via targeting of a nuclease-deficient form of SaCas9 (dSaCas9) fused to a Krüppel-associated box (KRAB) repressor to the *FAAH-OUT* promoter.^25,26^ When localized to DNA, dSaCas9-KRAB recruits a heterochromatin-forming complex that causes histone deacetylation and methylation (H3K9 trimethylation).^26,27^

**Figure 3:**
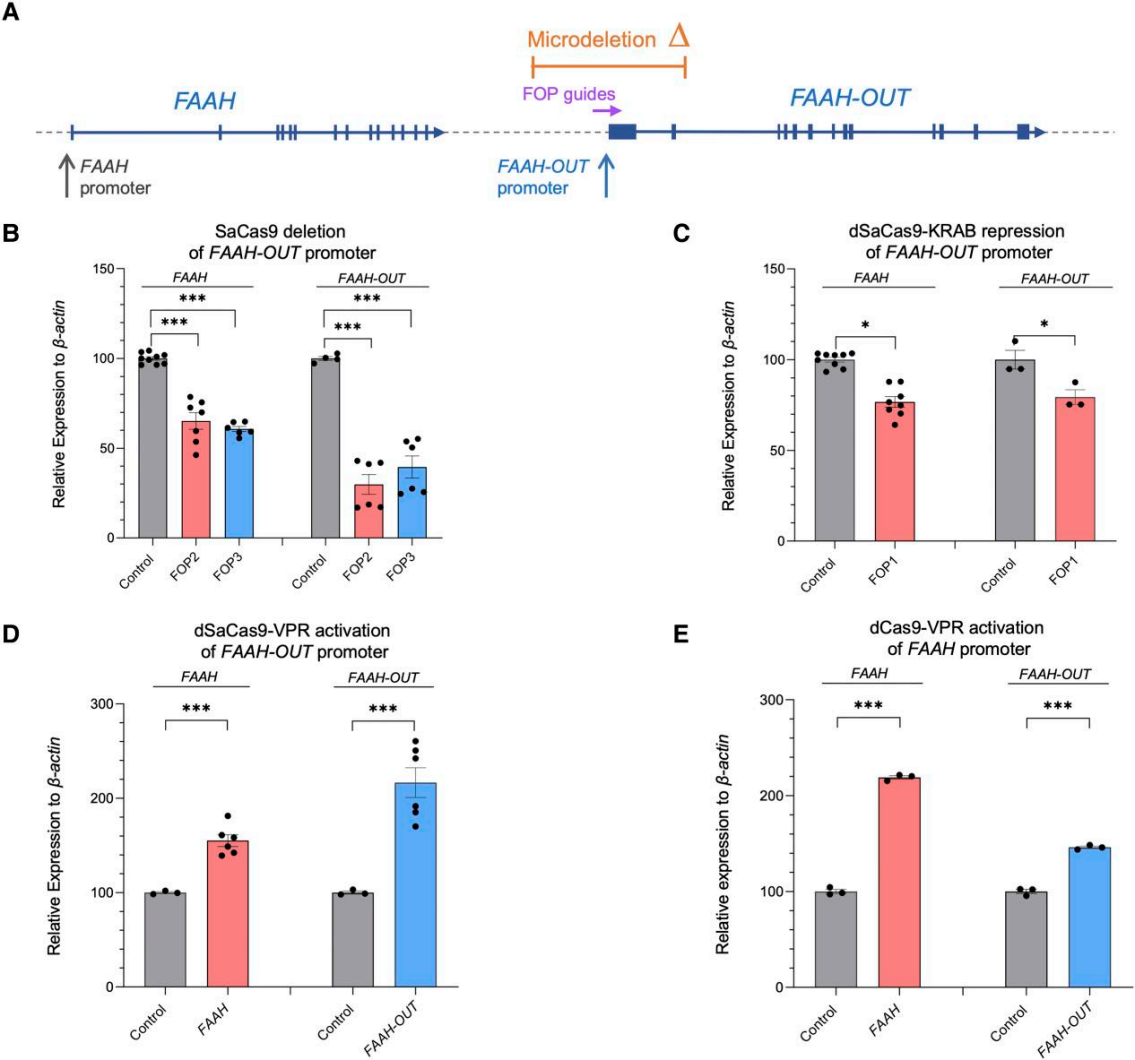
***FAAH-OUT* promoter modulates both *FAAH-OUT* and *FAAH* expression.** (**A**) Map showing relative positions of the ∼8 kb microdeletion identified in Patient PFS (in orange), *FAAH* and *FAAH-OUT* promoters, and FOP CRISPR guides (in purple) that map to the promoter region of *FAAH-OUT.* Exons are denoted by blue boxes and the direction of transcription shown by arrows. (**B**) CRISPR/Cas9 -induced deletion of *FAAH-OUT* promoter leads to reduction in both *FAAH-OUT* and *FAAH* expression. RT-qPCR analysis of both *FAAH* and *FAAH-OUT* mRNA levels showed significant reduction in both transcripts’ expression levels when HEK293 cells were transiently transfected with CRISPR/Cas9 constructs carrying either of the guide RNA pairs: FOP2 (in red) or FOP3 (in blue) designed to delete the *FAAH-OUT* promoter. (**C**) dSaCas9-KRAB-mediated repression of *FAAH-OUT* promoter leads to reduction in both *FAAH-OUT* and *FAAH* expression. RT-qPCR analysis of both *FAAH* and *FAAH-OUT* mRNA levels showed significant reduction after dSaCas9-KRAB-mediated repression of the *FAAH-OUT* promoter using FOP1 guide RNA in HEK293 cells. (**D**) dSaCas9-VPR-mediated activation of *FAAH-OUT* promoter leads to increase in both *FAAH-OUT* and *FAAH* expression. RT-qPCR analysis of *FAAH* and *FAAH-OUT* mRNA levels showed significant increase in both *FAAH* and *FAAH-OUT* transcript levels after targeted transcriptional activation of *FAAH-OUT* promoter using dSaCas9-VPR in HEK293 cells. dSaCas9-VPR-mediated *FAAH-OUT* activation led to transcriptional upregulation of *FAAH* gene when compared to control (empty vector). (**E**) dCas9-VPR-mediated activation of *FAAH* promoter leads to increase in both *FAAH* and *FAAH-OUT* expression. RT-qPCR analysis of *FAAH* and *FAAH-OUT* mRNA levels showed significant increase in both *FAAH* and *FAAH-OUT* transcript levels after targeted transcriptional activation of *FAAH* promoter using dCas9-VPR in HEK293 cells. dCas9-VPR-mediated activation of *FAAH* also led to upregulation of *FAAH-OUT* transcript levels. In all experiments the normalized expression value of control (relevant empty vector) was set to 100, and all other gene expression data were compared to that sample. Data-points are denoted by dots, bars show the ± SEM, and data analysed by a Student’s *t*-test, *P ≤ 0.05, **P ≤ 0.01, ***P ≤ 0.001.

Guide-pair RNA sequences (Fig. 3A and Supplementary Table 1) were selected to delete the *FAAH-OUT* promoter and cloned into an SaCas9-expressing vector. HEK293 cells were transiently transfected and the activity of each sgRNA-pair was assessed 72 h after transfection by RT-qPCR for *FAAH* and *FAAH-OUT* mRNA expression. Both *FAAH* and *FAAH-OUT* had markedly reduced expression when the FOP2- and FOP3-guide pairs were used to induce a deletion in the *FAAH-OUT* promoter compared to cells transfected with SaCas9 only (Fig. 3B). Similarly, epigenetic silencing of the *FAAH-OUT* promoter using the FOP1 sgRNA, which is located ∼330 bp upstream of the transcriptional start site previously identified by 5’RACE, led to a significant reduction in *FAAH-OUT* and *FAAH* expression levels (Fig. 3C). These results suggest that transcription of *FAAH-OUT* contributes to normal expression levels of *FAAH* and its product possibly acts as an enhancer lncRNA, similar to how *lincRNA-Cox2* functions to regulate the upstream *Ptgs2* gene.^28^

To further investigate whether *FAAH-OUT* can function as an enhancer lncRNA, we employed the CRISPR activation (CRISPRa) system to recruit a strong transcriptional activator to the *FAAH-OUT* putative promoter region and activate the gene in *cis*. We successfully increased *FAAH-OUT* expression more than 2-fold in transiently transfected HEK293 cells which lead to a ∼60% increase in *FAAH* expression, as measured by RT-qPCR (Fig. 3D). The reciprocal CRISPR activation of the *FAAH* promoter led to a more than 2-fold increase in *FAAH* mRNA levels and a ∼50% rise in *FAAH-OUT* expression (Fig. 3E), suggesting that transcription regulation of *FAAH* and *FAAH-OUT* within this locus is interconnected.

### Highly conserved ‘FAAH-AMP’ element functions as an enhancer for *FAAH* expression

**Figure 4:**
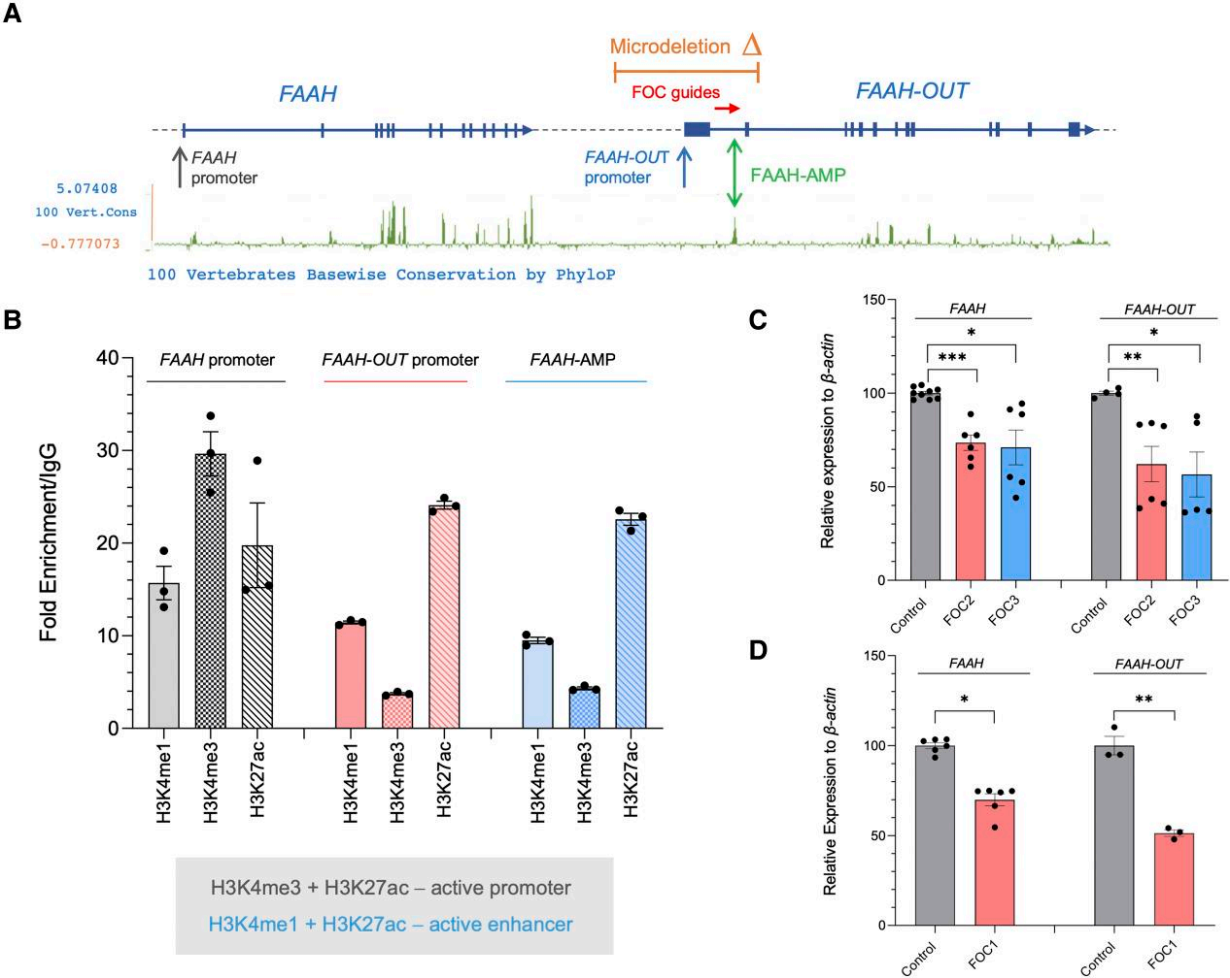
**Highly conserved ‘FAAH-AMP’ element regulates both *FAAH-OUT* and *FAAH* expression.** (**A**) Map showing relative positions of the ∼8 kb microdeletion identified in patient PFS (in orange), *FAAH* and *FAAH-OUT* promoters, ‘FAAH-AMP’ conserved element (double green arrow) and FOC CRISPR guides (red arrow) that map to the FAAH-AMP sequence. Exons are denoted by blue boxes and the direction of transcription shown by arrows. The PhyloP base-wise conservation track for 100 vertebrates from the UCSC genome browser shows regions of high sequence conservation as peaks (in green), with the majority of these mapping to gene exons in *FAAH* and *FAAH-OUT*. (**B**) Histone modification markers at FAAH-AMP, *FAAH* and *FAAH-OUT* promoters. ChIP-qPCR analysis at *FAAH* promoter demonstrated an enrichment of H3K4me3 and H3K27ac post-translational modifications that typically correlate with active promoters. Both *FAAH-OUT* promoter region and FAAH-AMP element sequence were enriched in H3K4me1 and H3K27ac post-translational modifications that typically correlate with active enhancers. (**C**) CRISPR/Cas9-induced deletion of FAAH-AMP leads to reduction in both *FAAH-OUT* and *FAAH* expression. RT-qPCR analysis of both *FAAH* and *FAAH-OUT* mRNA levels showed significant reduction in both transcripts’ expression levels when HEK293 cells were transiently transfected with CRISPR/Cas9 constructs carrying either of guide RNA pairs: FOC2 (in red) or FOC3 (in blue) designed to delete the FAAH-AMP conserved element. (**D**) dSaCas9-KRAB-mediated repression of FAAH-AMP leads to reduction in both *FAAH-OUT* and *FAAH* expression. RT-qPCR analysis of both *FAAH* and *FAAH-OUT* mRNA levels showed significant reduction after dSaCas9-KRAB-mediated repression of the FAAH-AMP conserved element using FOC1 guide RNA in HEK293 cells. The normalized expression value of control (relevant empty vector) was set to 100 and all other gene expression data were compared to that sample. Data-points are denoted by dots, bars show the ± SEM, and data analysed by a Student’s *t*-test, *P ≤ 0.05, **P ≤ 0.01, ***P ≤ 0.001.

Comparative genomic analyses across species can help to identify evolutionarily conserved sequences that may have important functions.^29^ By analysing the PhyloP basewise conservation track for 100 vertebrates on the UCSC genome browser, a highly conserved element (denoted ‘FAAH-AMP’) was identified in the first intron of *FAAH-OUT* (Fig. 4A). We considered that this region may contain important regulatory sequences for *FAAH-OUT* and/or *FAAH* expression.

Several studies have shown that active enhancer regions are enriched in specific histone modifications including histone 3 lysine 4 (H3K4) methylation and histone 3 lysine 27 (H3K27) acetylation^30-32^ and are highly conserved across species.^33^ We tested whether there are any enhancer marks present within the FAAH-AMP conserved region by ChIP-qPCR of H3K4 and H3K27 histone marks typically associated with enhancers and promoters, including H3K4 mono-methylation (H3K4me1), H3K4 tri-methylation (H3K4me3) and H3K27 acetylation (H3K27Ac).

By comparing immunoprecipitated chromatin DNA using primers targeting either the putative FAAH-AMP enhancer region or *FAAH-OUT* putative promoter with a gene desert control region by ChIP-qPCR, we observed that both the FAAH-AMP region and the *FAAH-OUT* upstream region showed strong enrichment in H3K27ac and H3K4me1 and a low level of H3K4me3 (Fig. 4B), a combination of post-translational modifications that is typically found at active enhancers.^34,35^ In contrast, the *FAAH* promoter region was enriched for H3K4me3 in keeping with typical active promoter-associated histone marks (Fig. 4B). The data therefore indicated that the FAAH-AMP conserved region indeed may function as an enhancer, potentially for *FAAH* expression.

To further test the functional importance of the FAAH-AMP conserved region as an enhancer element, we used CRISPR-Cas9 to delete the DNA containing the entire conserved FAAH-AMP region (Fig. 4A). Targeting of SaCas9 to the FAAH-AMP region by either of two independent set of guide RNAs (FOC2 and FOC3; Supplementary Table 1) achieved comparable and significant reductions in *FAAH* mRNA levels confirming that the FAAH-AMP region plays a positive regulatory role for *FAAH* gene expression (Fig. 4C).

Similarly, a reduction (∼30%) in *Faah* expression was also observed when the murine Faah-AMP region was deleted following transient transfection of mouse CAD cells with SaCas9 and the FOC4 guide-pair (Supplementary Fig. 4 and Supplementary Table 1).

Next, we used a guide sequence (FOC1) to recruit dSaCas9-KRAB to FAAH-AMP to enforce inhibition of the region’s regulatory elements without cutting out the FAAH-AMP sequence. Upon transient expression of the FOC1 sgRNA with CRISPRi in HEK293 cells, we observed significant repression of *FAAH* gene expression compared to control (Fig. 4D). Taken together these results indicate that the FAAH-AMP region contains an enhancer element that contributes to normal *FAAH* expression, and this regulatory mechanism appears to be conserved between different species. Interestingly, bioinformatic analysis of the FAAH-AMP sequence across species together with ChIP-Seq data analyses show that FAAH-AMP is a hub for transcription factor binding (Supplementary Fig. 4), further explaining its importance as an enhancer element.

### *FAAH-OUT* transcription modulates *FAAH* promoter methylation

Disruption of *FAAH-OUT* transcription, either by induced promoter deletion or epigenetic inhibition, leads to reduced *FAAH* expression (Fig. 3). This can be explained by either limited access to the FAAH-AMP enhancer region and/or a potential regulatory role for the *FAAH-OUT* lncRNA. There are several reports of regulatory lncRNAs that facilitate the status of target promoter and/or enhancer regions, such as recruiting chromatin remodellers, transcription factors and DNA modifiers such as DNA methylases or DNA demethylases.^36-39^ Considering that the *FAAH* promoter has a strong and conserved CpG island, modulating DNA methylation status in order to regulate *FAAH* expression is a possibility. Furthermore, the *FAAH* gene region sequence has been reported to have DNMT1-dependent DNA methylation.^37^

CpG-rich promoters are typically unmethylated, marked with histone modifications such as H3K4me3, and are highly active. If the *FAAH-OUT* lncRNA normally regulates levels of DNA methylation at the *FAAH* promoter, then loss of one *FAAH-OUT* allele (like in Patient PFS) could be sufficient to shift the balance of DNA methylation and chromatin modification towards *FAAH* promoter inactivation.

**Figure 5:**
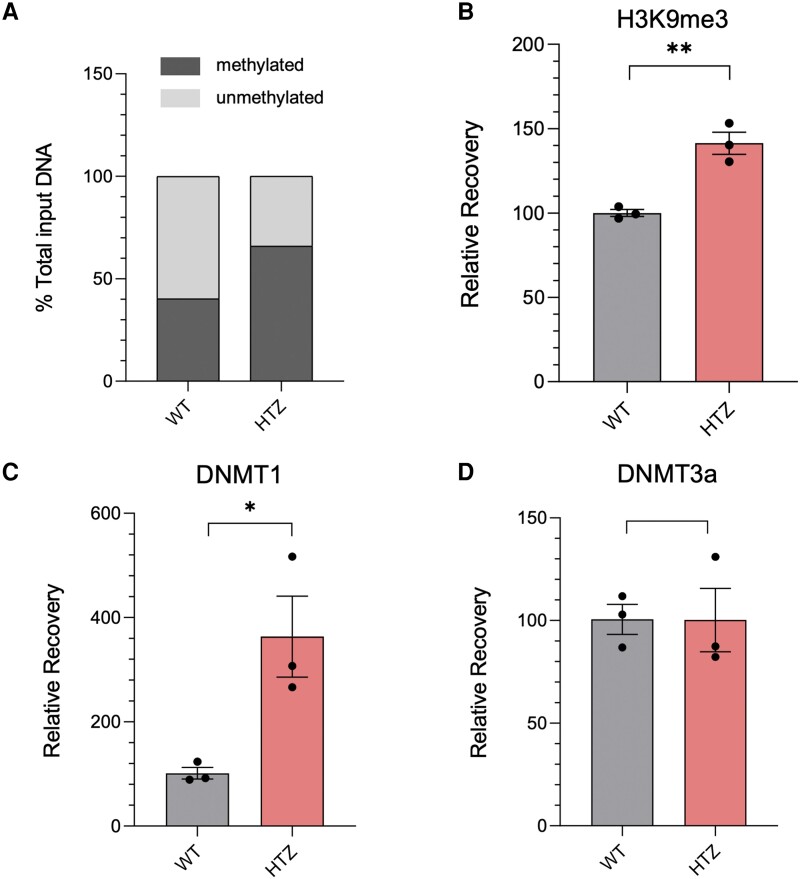
***FAAH* expression is regulated by DNA methylation in a DNMT1− and *FAAH-OUT*-dependent manner.** (**A**) *FAAH* promoter methylation is increased when *FAAH-OUT* levels are reduced. MethylScreen analysis showed a significant increase in *FAAH* promoter DNA methylation levels in HEK293-HTZ stable line cells harbouring heterozygous (HTZ) *FAAH-OUT* microdeletion (∼40% methylation in WT cell line to ∼65% methylation in HTZ line). (**B**) *FAAH* promoter is less active when *FAAH-OUT* transcription is reduced. ChIP-qPCR analysis at *FAAH* promoter showed that the increased DNA methylation at the *FAAH* promoter in HEK293-HTZ cells coincides with an increase in H3K9me3 post-translational modification that typically correlates with heterochromatin, both indicating a less active *FAAH* promoter state. (**C** and **D**) DNA at *FAAH* promoter is methylated by DNMT1. ChIP-qPCR analysis at *FAAH* promoter in HEK293-HTZ cells showed a significant (∼3-fold) increase in chromatin-associated DNMT1 (**C**) when compared to the wild-type (WT) control, whereas the levels of chromatin-bound DNMT3a did not change (**D**). Mean values from the WT control cells were assigned as 100%. Data-points are denoted by dots, bars show the ± SEM, and data analysed by a Student’s *t*-test, *P ≤ 0.05, **P ≤ 0.01 (**B**–**D**).

To test whether the reduction in *FAAH-OUT* expression affects the local epigenomic profile at the *FAAH* promoter region, we used DNA methylation and ChIP-qPCR assays to screen for levels of methylated DNA at the *FAAH* promoter in a heterozygous HEK293 *FAAH-OUT*^+/−^ cell line. As shown in Fig. 5A, in normal wild-type (WT) cells, methylation levels at the *FAAH* promoter are balanced between 40% methylated and 60% unmethylated DNA, with methylation rising sharply by ∼60% in heterozygous (HTZ) cells with reduced levels of *FAAH-OUT* expression, reversing the methylated versus unmethylated ratio. This suggests that lower levels of the *FAAH-OUT* lncRNA due to one allele loss leads to local epigenetic changes that drive *FAAH* expression down. Furthermore, the epigenetic inactivation of the *FAAH* promoter is enhanced by a rise in H3K9me3 modification (Fig. 5B).

We next explored whether this enhanced DNA methylation at the *FAAH* promoter in heterozygous HEK293 *FAAH-OUT*^+/−^ cells is provided by one of the known DNA methylases. ChIP-qPCR analysis showed that DNMT1 localization at the *FAAH* promoter was enriched 3-fold, whereas levels of DNMT3a did not change (Fig. 5C and D). This indicates that loss of the *FAAH-OUT* allele and/or reduction in *FAAH-OUT* lncRNA levels lead to increased recruitment of DNMT1 to the *FAAH* promoter and a rise in DNA methylation, in keeping with previously reported data for DNMT1-dependent genome-scale methylation profiling.^37^

### Transcriptomic analyses of Patient PFS-derived fibroblasts

**Figure 6:**
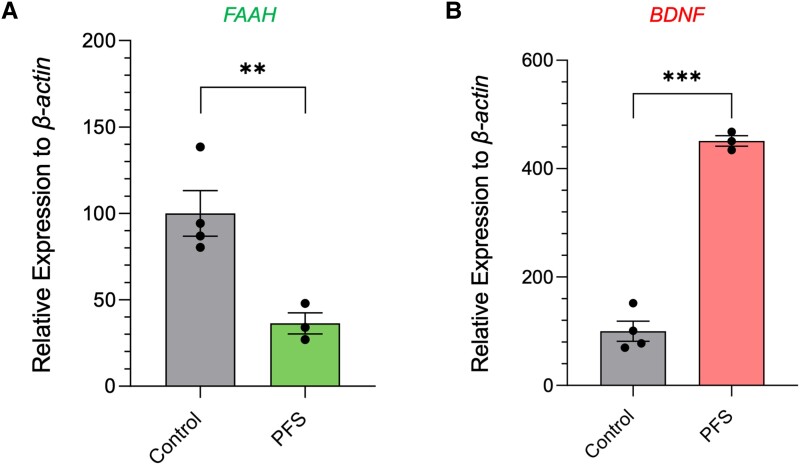
**Differential gene expression in cells with *FAAH-OUT* microdeletion.** (**A**) *FAAH* mRNA levels are reduced in patient fibroblasts. RT-qPCR analysis of *FAAH* mRNA levels in Patient PFS-derived fibroblasts showed significant reduction in *FAAH* mRNA levels when compared to four gender matched controls. (**B**) *BDNF* mRNA levels rise in patient fibroblasts. RT-qPCR analysis of *BDNF* mRNA levels in Patient PFS-derived fibroblasts showed a significant rise in *BDNF* mRNA levels when compared to the four gender matched controls. For **A** and **B**, data were normalized to the beta-actin gene as an endogenous control. The normalized expression value of control subjects was set to 100. Data-points are denoted by dots, bars show the ± SEM, and data analysed by Student’s *t*-test, **P ≤ 0.01, ***P ≤ 0.001.

Primary fibroblast cell lines derived from Patient PFS and four unrelated female healthy controls were cultured and total RNA isolated. *FAAH* expression, as shown by RT-qPCR, was significantly downregulated in the patient-derived fibroblast cell line compared to controls (Fig. 6A). To explore whether additional genes were also dysregulated and to identify potential downstream candidate genes and pathways that could help explain the Patient PFS phenotype, we carried out a whole transcriptome microarray. This showed striking gene dysregulation (Table 1, Supplementary Fig. 5A and Supplementary Table 5) with 797 genes upregulated and 348 genes downregulated (>2-fold change; *P* < 0.05) between the Patient PFS line and four control subjects. Ingenuity Pathway analyses highlighted groups of gene products which take part in WNT-induced signalling, wound-healing, brain-derived neurotrophic factor (BDNF)-signalling and G-protein signalling (Supplementary Fig. 5B). A number of genes connected to WNT-regulated pathways were dysregulated included the downregulated stimulators of canonical WNT-dependent pathway *SFRP2* and *SERPINF1*, the upregulated repressor *DKK1*, and the upregulated *WNT5B* and *WNT16* transcription factors (Table 1 and Supplementary Fig. 6).^40-45^

**Table 1:** Differential gene expression in cells with *FAAH-OUT* microdeletion

Key DEGs	Fold change	*P*-value
*DKK1*	40.19	0.0073
*GABBR2*	31.06	0.0001
*BDNF*	10.06	0.0011
*WNT5B*	3.95	0.0254
*WNT16*	3	0.0024
*ACKR3*	−11.45	0.032
*SERPINF1*	−53.4	0.0015
*SFRP2*	−321.2	0.0001

Fold change in expression of key differentially expressed genes (DEGs) in Patient PFS-derived fibroblast cell line compared to gender matched controls (microarray analyses).

One gene of interest that was significantly upregulated in the PFS cell line was *BDNF*, with the microarray assay result validated by RT-qPCR (Fig. 6B). Interestingly, previous work in rats has shown that pharmacological inhibition of the FAAH enzyme elevated BDNF levels.^46-48^ We replicated these data in wild-type mice by showing that systemic injection of FAAH inhibitor URB597 showed a ∼25% increase in hippocampal BDNF levels, as determined by ELISA (Supplementary Fig. 7A). The connection between loss of FAAH activity and increased BDNF levels is particularly interesting given the patient’s reported elevated mood and the known anti-depressive actions of BDNF and TrkB signalling.^21,49^

Another gene of interest that is significantly downregulated in the Patient PFS cell line encodes the atypical chemokine receptor *ACKR3*, with the microarray result validated by RT-qPCR (Supplementary Fig. 6E). ACKR3 is a broad-spectrum opioid scavenger receptor, downregulation of which could contribute to the painless phenotype.^50-52^ We confirmed the connection between *FAAH* downregulation and *ACKR3* transcript levels by using silencing RNA against *FAAH* in a HEK293 cell line, which led to a ∼40% decrease in *ACKR3* levels (Supplementary Fig. 7B).

Patient PFS has previously observed that wounds heal quickly and work carried out in mice has shown that genetic or pharmacological inhibition of FAAH activity accelerates skin wound healing.^53^ We analysed cell migration of the Patient PFS fibroblasts compared to control fibroblasts using a scratch assay and time-lapse microscopy, which showed that gap closure was significantly faster in the Patient PFS fibroblasts (Supplementary Fig. 8). This is consistent with previous work where human keratinocytes also showed a marked increase in migration in a scratch assay in the presence of a FAAH inhibitor and supports FAAH as a potential therapeutic target for wound healing.^53^

## Discussion

In this study we provide the first mechanistic insights into how the microdeletion identified in Patient PFS negatively affects *FAAH* expression and leads to pain insensitivity, accelerated wound healing and the lack of depression and anxiety symptoms observed in the patient. The ∼8 kb microdeletion contains the upstream promoter region and first two exons of *FAAH-OUT* and also an evolutionary conserved ‘FAAH-AMP’ element in the first intron that contains several potential transcription factor binding sites. We show that FAAH-AMP has the chromatin marks of an active enhancer and positively regulates *FAAH* expression. CRISPR interference at the FAAH-AMP enhancer element or the *FAAH-OUT* promoter results in reduced levels of both *FAAH-OUT* and *FAAH* mRNA in human cells, indicating that *FAAH* expression is regulated by transcriptional activity at the *FAAH-OUT* locus. The FAAH-AMP enhancer element is also conserved in mice, with CRISPR editing similarly leading to a reduction in *Faah* expression. Furthermore, we show that *FAAH* and *FAAH-OUT* are co-expressed within the same cells, with the *FAAH-OUT* lncRNA being enriched in nuclei as shown by RNAscope experiments in different human tissues and cell fraction analyses.

**Figure 7:**
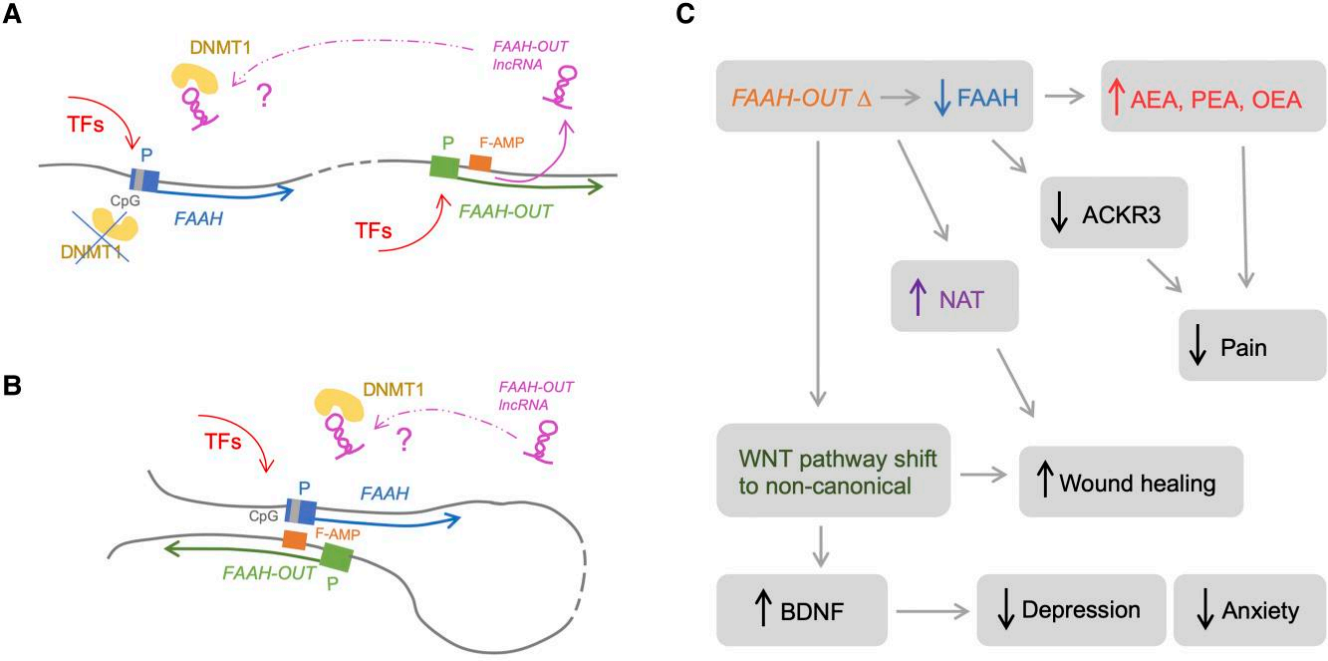
**Schematic representation of *FAAH-OUT*—dependent regulation of *FAAH* expression and subsequent phenotypical changes in the patient**. (**A**) *FAAH-OUT* transcription regulates *FAAH* expression via preventing DNMT1-dependent DNA methylation at *FAAH* promoter. Activation of *FAAH-OUT* promoter (in green) leads to transcription of *FAAH-OUT* lncRNA (in purple) and opening of chromatin at FAAH-AMP (F-AMP, in orange)*. FAAH-OUT* lncRNA potentially prevents DNMT1 (in yellow) recruitment to methylate *FAAH* promoter (in blue) at CpG island (in grey) allowing *FAAH* gene to be transcribed at higher levels. (**B**) Additional level of regulation via FAAH-AMP enhancer (F-AMP, in orange) could be provided via potential looping between the enhancer and *FAAH* promoter in blue to facilitate transcription factors (TFs) binding. (**C**) Schematic network of key dysregulated genes and pathways that result from the disruption of the *FAAH − FAAH-OUT* axis, providing molecular basis for the phenotypes observed in the patient. Microdeletion in *FAAH-OUT* leads to reduction in *FAAH* expression and subsequent fall in overall FAAH activity thus leading to rise in endocannabinoid levels (AEA, PEA and OEA), which (especially anandamide, AEA) facilitate pain insensitivity. The mutation also leads to a drop in ACKR3 levels, lack of which as a broad-spectrum scavenger for opioid peptides adds another potential level to the patient’s analgesia. In addition, the decrease in FAAH activity leads to a rise in *N*-acyl taurine (NAT) and changes in WNT pathways (shift from canonical to non-canonical) both of which likely contribute to accelerated wound healing. The WNT pathway shift also leads to a dramatic rise in BDNF levels, thus protecting the patient from depression and anxiety.

The data suggest two mechanisms of *FAAH-OUT*-dependent in *cis* regulation of *FAAH* in which transcription of the *FAAH-OUT* gene leads to (i) expression of the *FAAH-OUT* lncRNA that may play a role as a positive regulator of *FAAH*; and (ii) opening up of chromatin in the FAAH-AMP enhancer region, which improves accessibility to the region for proteins that in turn modulate efficiency of *FAAH* transcription and potentially allow local looping between *FAAH* and *FAAH-OUT* genes for co-ordinated transcription (Fig. 7A and B). The reduction in *FAAH-OUT* transcription leads to enhanced DNMT1-dependent DNA methylation of the CpG island within the *FAAH* gene promoter, and subsequent chromatin remodelling as witnessed by increased H3K9 trimethylation, resulting in transcriptional shutdown of *FAAH.* The *FAAH-OUT* lncRNA may therefore regulate *FAAH* expression via preventing DNMT1-dependent DNA methylation of the *FAAH* promoter, thus maintaining its transcriptional potential. DNMT1 methylation regulation of the *FAAH* promoter has previously been reported, as have examples of other lncRNAs that regulate DNA methylation at the promoter regions of other genes.^36-38^

Further work will help to understand exactly how *FAAH-OUT* may be functioning as an enhancer RNA and whether the *FAAH-OUT* lncRNA forms complexes directly at the *FAAH* promoter and/or FAAH-AMP region, similar to other known lncRNA transcriptional regulators.^36-39^ In addition to possibly protecting the *FAAH* promoter from DNMT1-dependent DNA methylation, the *FAAH-OUT* lncRNA may play a role in keeping the *FAAH* promoter active by maintaining an R-loop at that region. R-loops (three-stranded RNA/DNA structures) form when a nascent transcript or a lncRNA invades and makes a complex with a DNA duplex and are widespread at the GC-rich regions of promoters, protecting CpG islands from DNA methylation and preventing silencing.^54,55^ Both *FAAH* and *FAAH-OUT* promoters are GC-rich with >100 CpG pairs within the *FAAH* promoter sequence and about half of those are clustered in large CpG islands. R-loop formation could be achieved by locking RNA onto the DNA strand via 4xG repeats using a velcro-type interaction between them and quadruple Cs on the other strand.^56^ The *FAAH* promoter sequence has ∼10 4xGs whereas the *FAAH-OUT* lncRNA has 20 4xGs.

It is also of interest that chromatin marks at the *FAAH-OUT* promoter region were similar to those at the FAAH-AMP region and resembled those of an active enhancer (enriched in H3K27Ac and H3K4me1) rather than a promoter (enriched in H3K4me3 and H3K27Ac) (Fig. 4B). This suggests that either the *FAAH-OUT* promoter has a dual role serving as a promoter for *FAAH-OUT* and an enhancer for *FAAH* or it has been evolutionary repurposed from promoter to enhancer, thus modulating *FAAH* expression together with FAAH-AMP.^57,58^ This would be consistent with our data where transcriptional activation of *FAAH-OUT* via the CRISPRa system leads to an upregulation of *FAAH* and vice versa, where activation of *FAAH* transcription leads to an increase in *FAAH-OUT* RNA levels (Fig. 3D and E).

We further aimed to understand how decreased levels of FAAH and higher levels of anandamide (and other substrates) translate into the patient’s pain insensitivity syndrome, which is characterized by the absence of postoperative pain, painless burns and bone fractures, a happy, non-anxious disposition, fast wound healing, fear and memory deficits, and significant postoperative nausea and vomiting induced by morphine.^21^ In the original case report, we also noted that her dental surgeon observed, most unusually, that her saliva dissolves the fixative for a temporary denture after just 90 min.^21^ Interestingly, there is a recent report of lipidomic profile differences in the submandibular gland of FAAH knockout versus wild-type mice.^59^ It will be interesting to assay the salivary lipidomic profiles of Patient PFS in potential future studies.

FAAH inhibitors have yet to reach the clinic as analgesics,^60^ with a major clinical trial with the irreversible FAAH inhibitor PF-04457845 failing to induce effective analgesia in patients with pain due to osteoarthritis of the knee.^17^ Whether this is a compound specific problem and/or whether efficacy would be achieved for another pain disorder remains to be fully determined. FAAH inhibitors in rodent models are analgesic and genetic knockout of *Faah* in mice causes hypoalgesia, but not complete painlessness.^61-63^ Our results highlight the importance of the *FAAH-OUT* lncRNA to the human pain insensitive phenotype observed in Patient PFS. Long non-coding RNAs are known for their exquisite spatial and temporal sensitivity in regulating gene expression.^64,65^ In future research, we aim to determine at the single-cell level the specific cellular populations that are contributing to the symptoms observed in Patient PFS and how *FAAH-OUT* dynamically regulates *FAAH* expression in these cells. In particular, we plan to determine whether *FAAH-OUT* acts in *trans* to regulate gene expression at other genomic loci, similar to other lncRNAs such as *lincRNA-Cox2* and *Firre*.^28,66^

In this study, to narrow in on the key functional targets downstream of the *FAAH—FAAH-OUT* axis, we used microarray analysis of patient-derived fibroblasts to uncover a network of key molecular pathways and genes that become dysregulated as a result of disrupting *FAAH-OUT*. There were 797 genes upregulated and 348 genes downregulated (>2-fold change; *P* < 0.05) between the Patient PFS line and four gender-matched controls. Pathway analyses showed major changes in expression level of genes which take part in WNT-induced signalling, wound healing, BDNF-signalling and G-protein signalling (Supplementary Fig. 5B). Thus, several genes connected to WNT-regulated pathways included upregulation of the *DKK1* repressor and *WNT5B* and *WNT16* transcription factors and downregulation of canonical WNT-dependent pathway stimulators such as *SFRP2.* This combined indicates that the reduced FAAH levels and activity lead to WNT pathway(s) shift from a canonical to non-canonical type. Importantly, WNT pathways have been previously linked to wound healing and both upregulated *Wnt5b* and *Wnt16* have also been linked to bone regeneration.^44,45,67-69^ In addition to gene expression changes highlighted in Table 1 and Supplementary Fig. 5B, FAAH is known to degrade NATs, which are lipids implicated in regulation of skin wound healing, thus further helping to explain the accelerated healing phenotype observed for Patient PFS.^53^

Interestingly, WNT-dependent signalling has been previously reported to be connected to levels of BDNF, which modulates mood and is directly linked to anxiety and depression through TrkB receptor signalling.^49,70,71^ Furthermore, pharmacological inhibition of FAAH activity has been reported to lead to an increase in BDNF levels in rats.^46-48^ Our gene expression analyses in patient-derived fibroblasts show a significant upregulation in *BDNF* expression, although whether this is replicated in other patient tissues remains to be tested. Nevertheless, we have shown that in mice, pharmacological inhibition of the FAAH enzyme also upregulates hippocampal BDNF, providing further evidence for the FAAH-BDNF link.

Another gene that is significantly upregulated is *GABBR2* which encodes receptor subunit GABAB_2_ which forms an active heterodimeric complex with GABAB_1_ in the GABAB receptor.^72^ GABAB receptors are abundant in the brain, where they are localized in many neuronal cell types including interneurons and some glial cells.^72^ GABBR2 inhibits neuronal activity via G-protein coupled secondary messenger systems and its low levels were implicated in reduced analgesic effects of oxycodone.^73^ Furthermore, GABAB receptor knockout mice data indicate a role for GABAB receptors in nociception and anxiety, with GABAB knockout mice showing increased anxiety.^74,75^ Interestingly, low levels of GABBR2 expression were shown to be a valid biomarker for patients with chronic migraine.^76^ Thus high levels of GABBR2 expression could be consistent with the pain- and anxiety-free phenotype of Patient PFS.^21^

Among significantly downregulated genes, one of particular interest is *ACKR3* (Supplementary Figs 5 and 6). *ACKR3* encodes the atypical chemokine receptor ACKR3/CXCR7 that is widely expressed in brain. ACKR3 has recently been reported as a broad-spectrum scavenger for opioid peptides and has also been identified as a natural target of conolidine, a natural analgesic alkaloid.^50,51^ These properties potentially make ACKR3 an important and physiologically relevant contributor to Patient PFS painless phenotype. A reduction in *ACKR3* expression levels resulting from downregulation of the *FAAH-FAAH-OUT* axis could lead (via downregulation of *ACKR3*) to higher availability of endogenous opioid peptides for the classical opioid receptors (Fig. 7C).

In summary, our data show that microdeletion in *FAAH-OUT* disrupts transcription of the *FAAH-OUT* lncRNA and eliminates the enhancer sequence element FAAH-AMP, thus leading to deregulation of the *FAAH-FAAH-OUT* axis. We demonstrate that reduction in *FAAH-OUT* transcription leads to DNMT1-dependent DNA methylation of the CpG island within the *FAAH* gene promoter, resulting in transcriptional shutdown of *FAAH* and reduction of FAAH activity. Moreover, through microarray analysis of Patient PFS-derived fibroblasts we have uncovered a network of key molecular pathways and genes that become dysregulated as a result of disrupting *FAAH-OUT* such as a shift in WNT-dependent pathways towards non-canonical, a dramatic increase in *BDNF* and a decrease in *ACKR3* expression levels.

Whilst further experiments would be needed to elucidate the precise mechanism(s) by which the *FAAH-OUT* lncRNA regulates *FAAH*, our data provide a significant advance in understanding inter-pathway crosstalk resulting from lower FAAH activity and higher anandamide levels connecting, for the first time, major players from the endocannabinoid system with those of G-protein and opioid signalling. The data thus provide a coherent explanation for the pain insensitivity, lack of anxiety, faster wound-healing and other syndromic symptoms observed in the patient and form a platform for development of future gene and small molecule therapies. Given the current failure of small molecule inhibitors of FAAH as human analgesics, our findings validate *FAAH-OUT* regulation of *FAAH* as a new route to develop pain treatments.

## Supplementary Material

awad098_Supplementary_Data
