# Supplementary material for: Molecular basis of FAAH-OUT-associated human pain insensitivity
Source: Brain. 2023 May 24;146(9):3851–65. doi: 10.1093/brain/awad098 (PMC10473560; doi:10.1093/brain/awad098)
Supplement: awad098_Supplementary_Data [file awad098_supplementary_data.zip › brain-2022-02087-File010.pdf]

Suppl. Fig S1

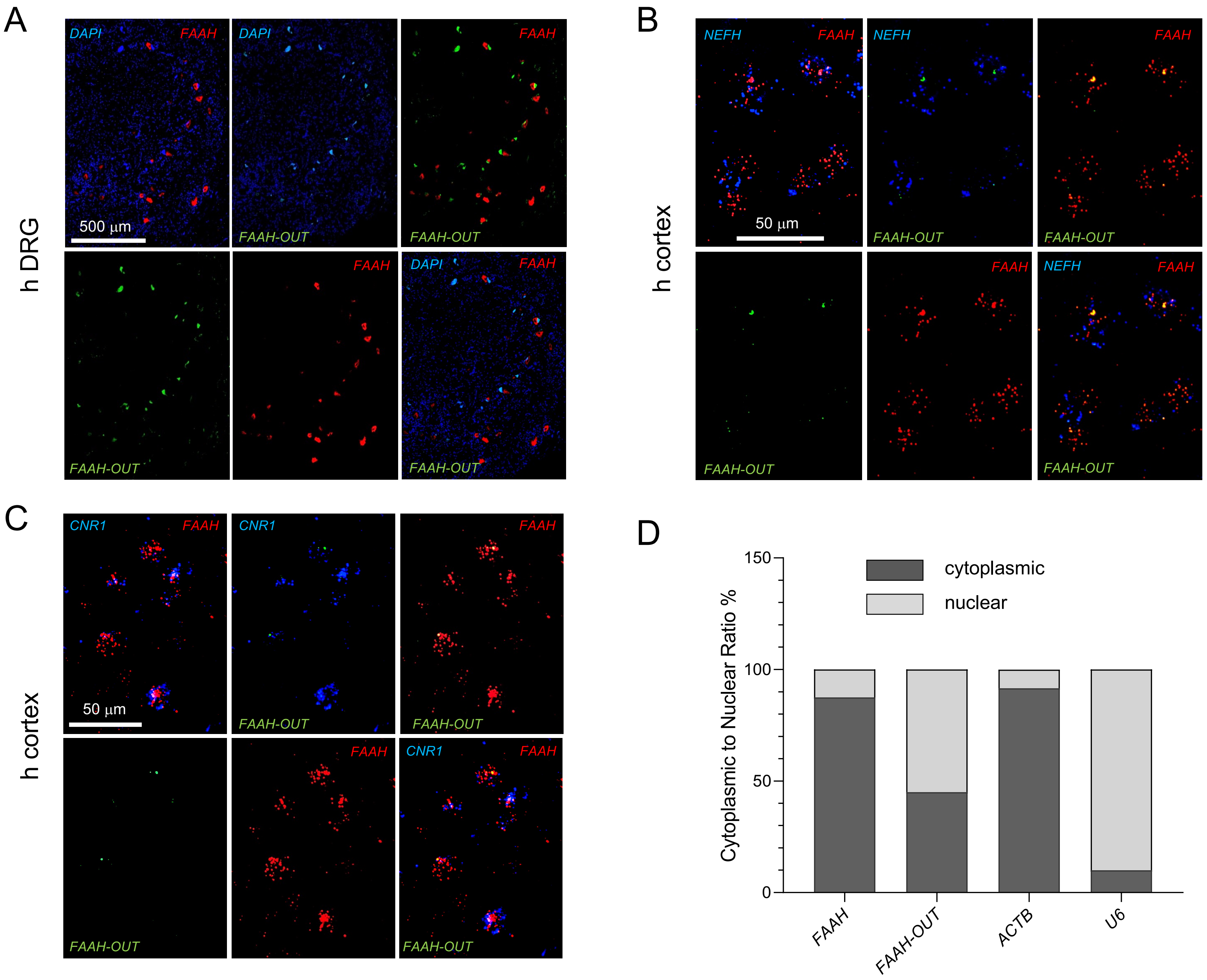

A
h DRG
DAPI
FAAH
FAAH-OUT
500 µm
B
h cortex
NEFH
FAAH
FAAH-OUT
50 µm
C
h cortex
CNR1
FAAH
FAAH-OUT
50 µm
D
Cytoplasmic to Nuclear Ratio %
cytoplasmic
nuclear
0
50
100
150
FAAH
FAAH-OUT
ACTB
U6

Supplementary Figure S1. *FAAH* and *FAAH-OUT* RNA expression levels and localisation.

(A*) FAAH* and *FAAH-OUT* RNA expression levels and localisation in human DRG neurons.
5 µM - thick cuts of human DRGs were analysed by RNAscope assay. Localisation of *FAAH-OUT* lncRNA (green, AF488) compared to *FAAH* mRNA (red, Opal570) as analysed by RNAscope assay in 5 µM - thick cuts of human DRGs. DAPI staining indicates nuclei (blue). Scale bars are shown in white.

(B and **C) *FAAH* and *FAAH-OUT* RNA expression levels and localisation in human cerebral cortex neurons**.
7-10 µM - thick fresh-frozen cuts of human cerebral cortex were fixed with 4% PFA and analysed by RNAscope assay. (**B)** – four representative cells show localisation of *FAAH-OUT* lncRNA (green, AF488) compared to *FAAH* mRNA (red, Opal570) and *NEFH* mRNA (blue, TS405) localisation. (**C**)– five representative cells show localisation of *FAAH-OUT* lncRNA (green, AF488) compared to *FAAH* mRNA (red, Opal570) and *CNR1* mRNA (blue, TS405) localisation. Scale bars are shown in white.

(D) Subcellular localisation of *FAAH* and *FAAH-OUT* RNAs.
RT-qPCR analysis of RNA following nuclear/cytoplasmic fractionation shows the distribution of the indicated transcripts in HEK293 cells. *FAAH* mRNA is predominantly cytoplasmic whereas *FAAH-OUT* lncRNA is enriched in the nucleus. *ACTB* and *U6* RNAs were used as cytoplasmic and nuclear controls respectively. The RT-qPCR data, represented as a percentage of the total amount of detected transcripts, are presented as mean of technical triplicates.

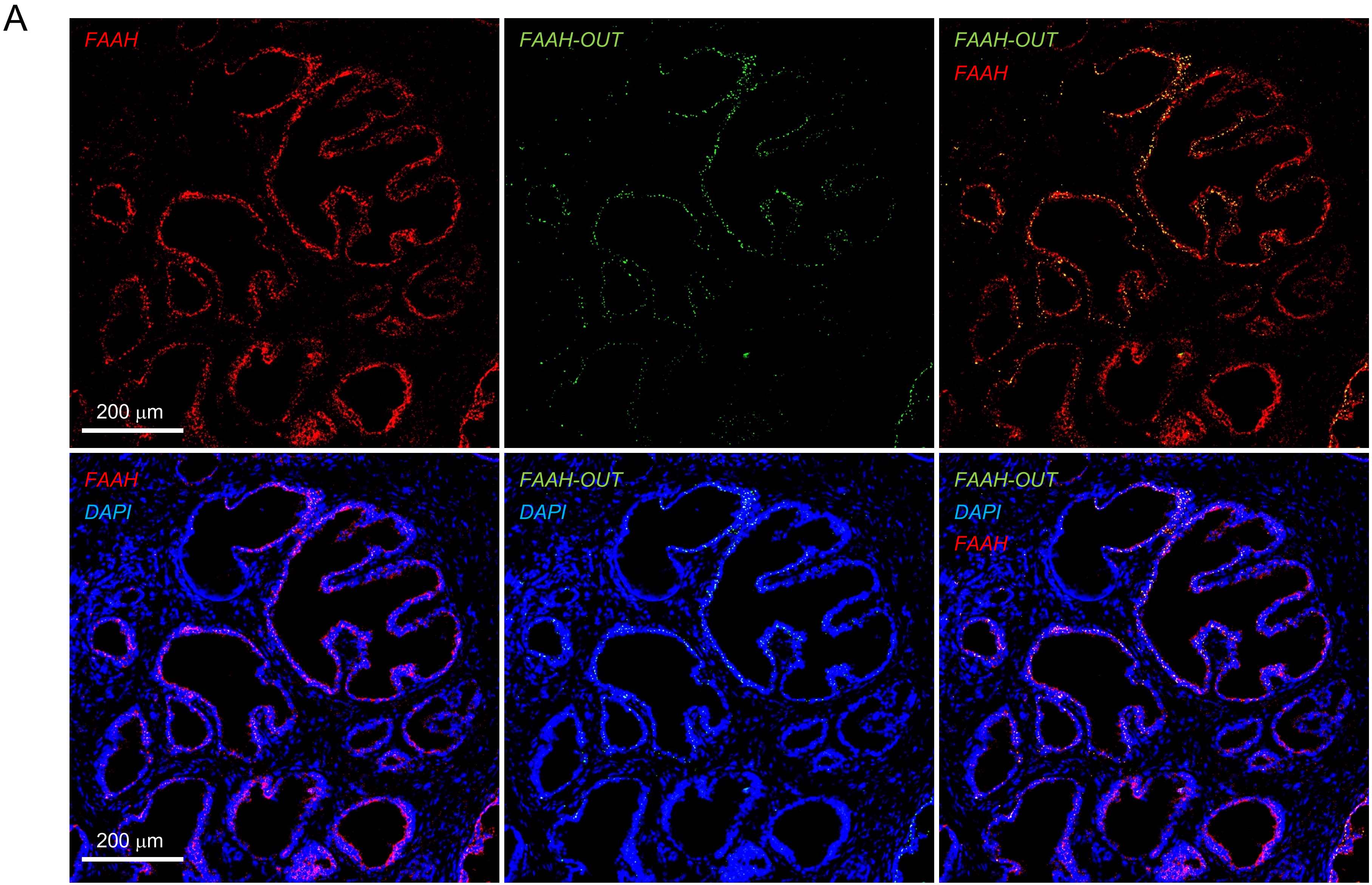

Supplementary Figure S2A. *FAAH* and *FAAH-OUT* RNA expression levels and localisation in human prostate tissue cells.
(**A**) 7-10 µM - thick cuts of fresh frozen prostate sections were analysed by RNAscope assay (Methods). Localisation of *FAAH-OUT* lncRNA (green, AF488) was compared to *FAAH* mRNA (red, Opal570) localisation and DAPI staining indicating nuclei positions (blue). Scale bars (200 mM) are in white.

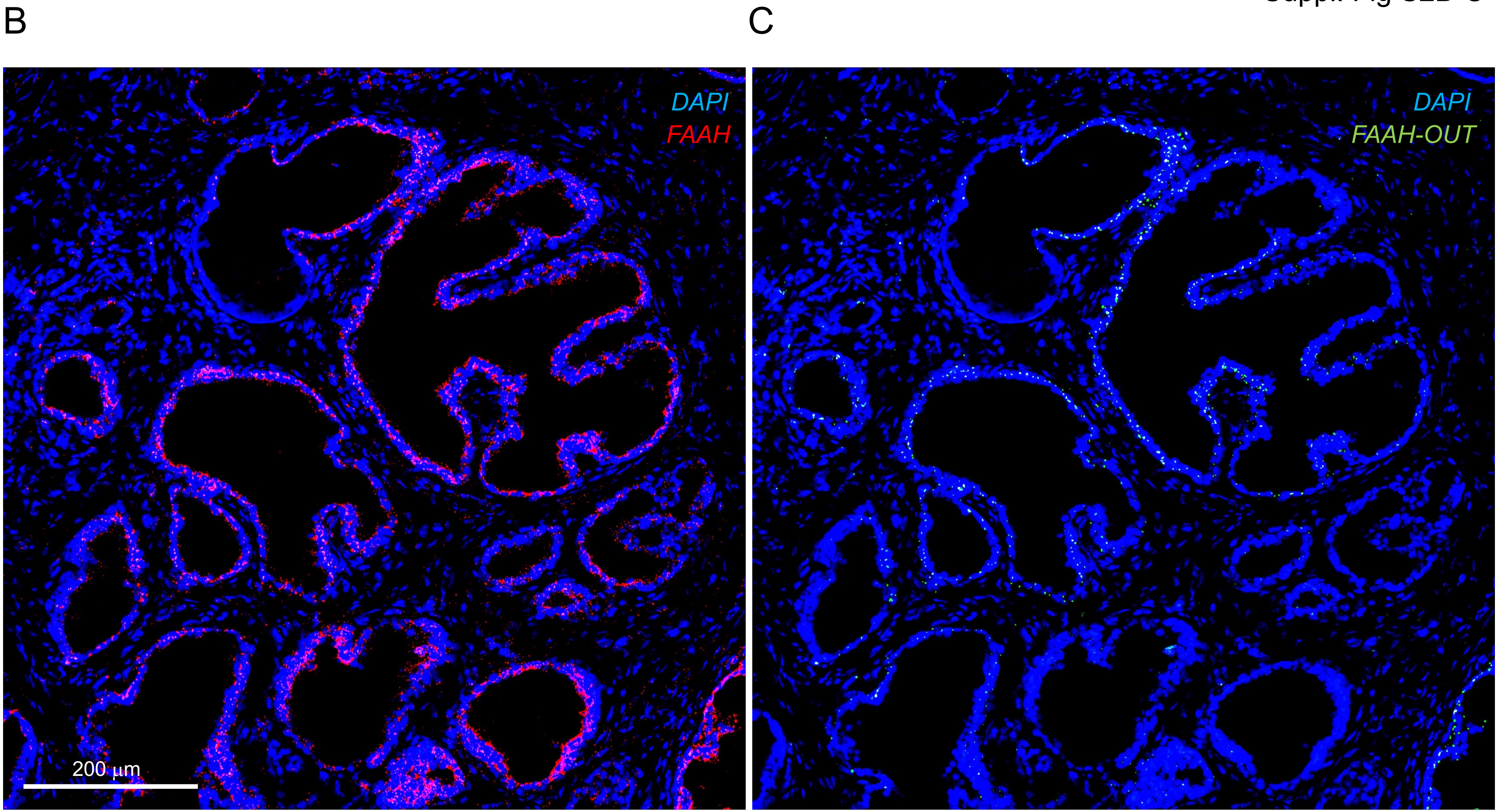

Supplementary Figure S2 (B and **C)** ***FAAH*** **and** ***FAAH-OUT*** **RNA expression levels and localisation in human prostate tissue cells.** **(B** and **C)** Enlarged views of prostate tissue section showing *FAAH* vs DAPI and *FAAH-OUT* vs DAPI and demonstrating mostly nuclear localisation for *FAAH-OUT* lncRNA and mostly cytoplasmic localisation for *FAAH* mRNA. Scale bar (200 mM) is in white.

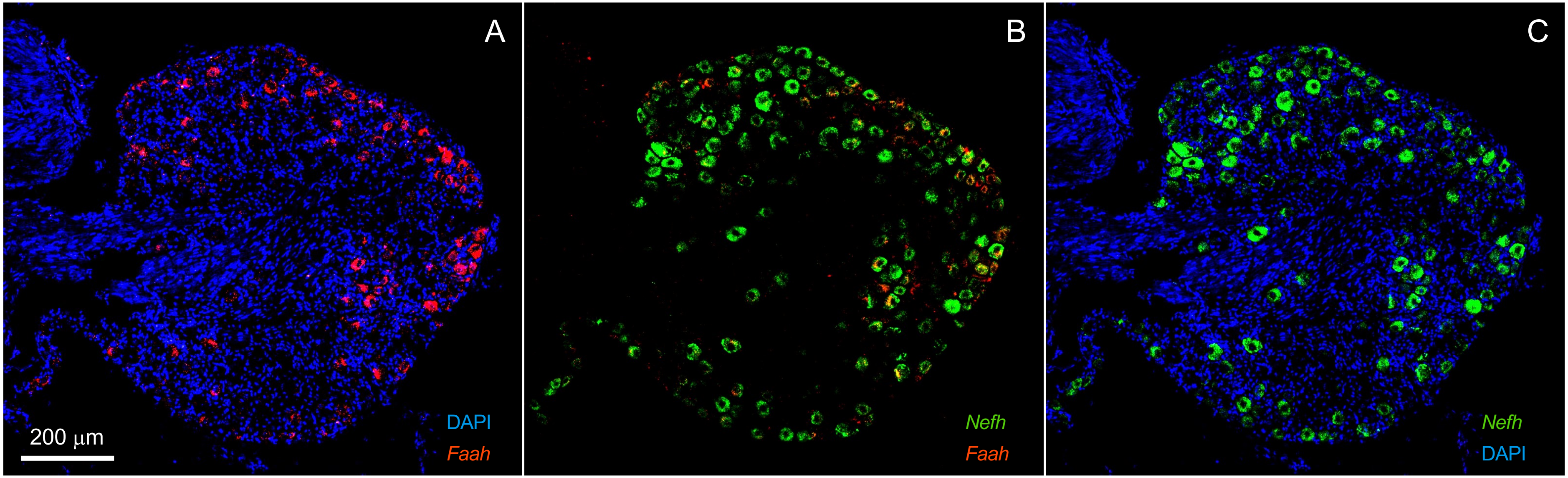

Supplementary Figure S3. *Faah* RNA expression levels and localisation in mouse DRG

Faah RNA expression levels and localisation in mouse DRG neurons. 11 µM - thick fresh-frozen cuts of mouse lumbar DRGs were fixed with 4% PFA and analysed by RNAscope assay. (**A-C**). Localisation of *Faah m*RNA (**A**, red, Opal570) compared to *Nefh* mRNA (**B** and **C,** green, AF488) localisation and DAPI staining indicating nuclei positions (**A** and **C**, blue). Scale bar (200 mM) is shown in white.

Suppl. Fig S4A

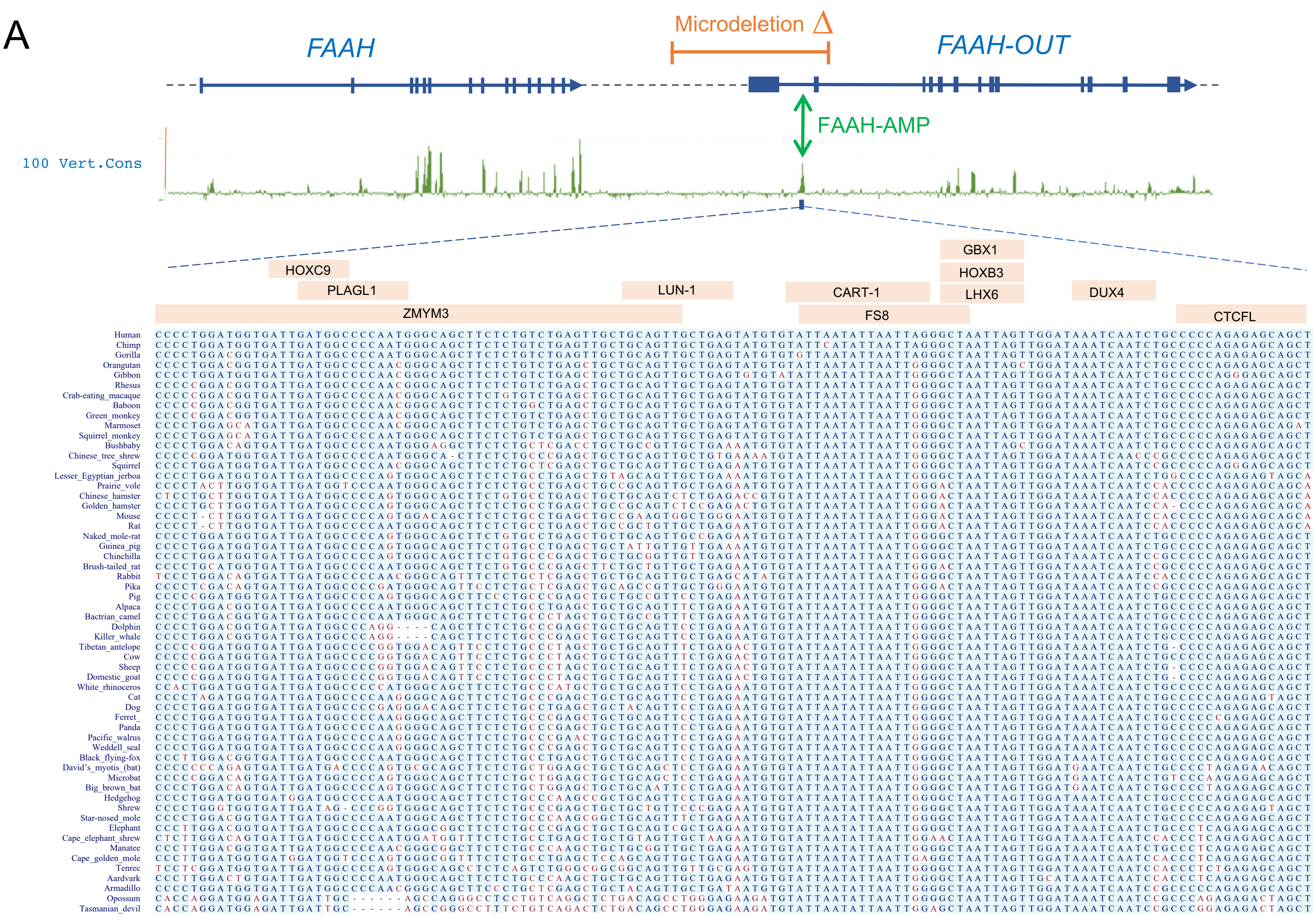

Supplementary Figure S4A. FAAH-AMP is highly conserved among species. **(A)** Map showing relative positions of the ~8 kb microdeletion identified in patient PFS (in orange) and 'FAAH-AMP' conserved element indicated with green double arrow. Exons are denoted by blue boxes and the direction of transcription shown by arrows. The PhyloP base-wise conservation track for 100 vertebrates from the UCSC genome browser shows regions of high sequence conservation as peaks (in green), with the majority of these mapping to gene exons in *FAAH* and *FAAH-OUT*. The core of the conserved element (chr1:46,424,925-46,425,054; build hg38) is expanded in multiple alignment of the representative vertebrates. The positions of DNA-binding for selected human transcription factors (TFs) are indicated according to Transfac and Jaspar databases.

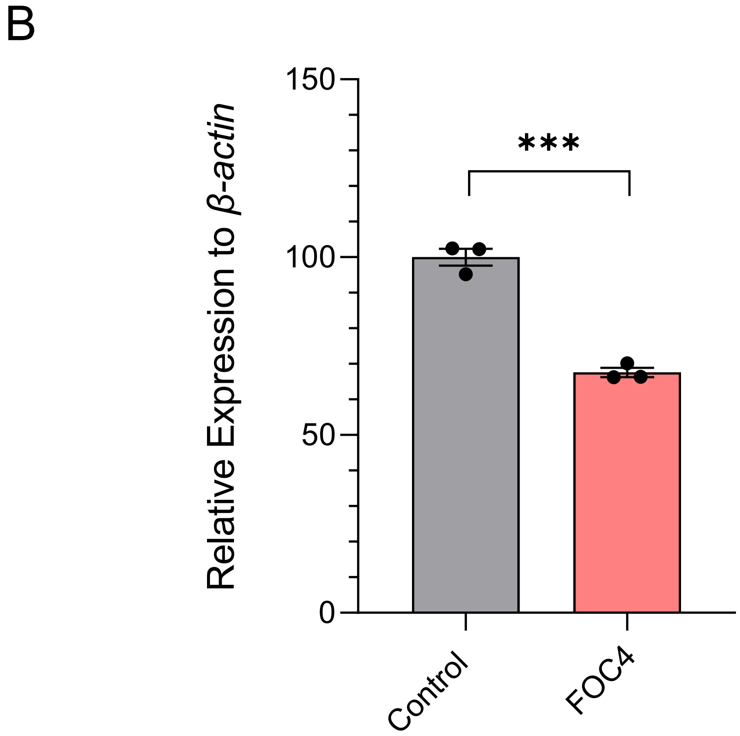

Supplementary Figure S4B. CRISPR/Cas9-induced deletion of Faah-AMP leads to reduction in *Faah* expression in mouse cells.

(**B**) RT-qPCR analysis of *Faah* mRNA levels showed significant reduction in its levels when mouse CAD were transiently transfected with CRISPR/cas9 constructs with guide RNA pairs: FOC4 designed to delete the Faah-AMP conserved element. The normalized expression value of control (empty vector) was set to 100 and all other gene expression data were compared to that sample. Data points are denoted by dots, bars show the ± SEM, and data analysed by Student's t-test, *** $P < 0.001$.

A

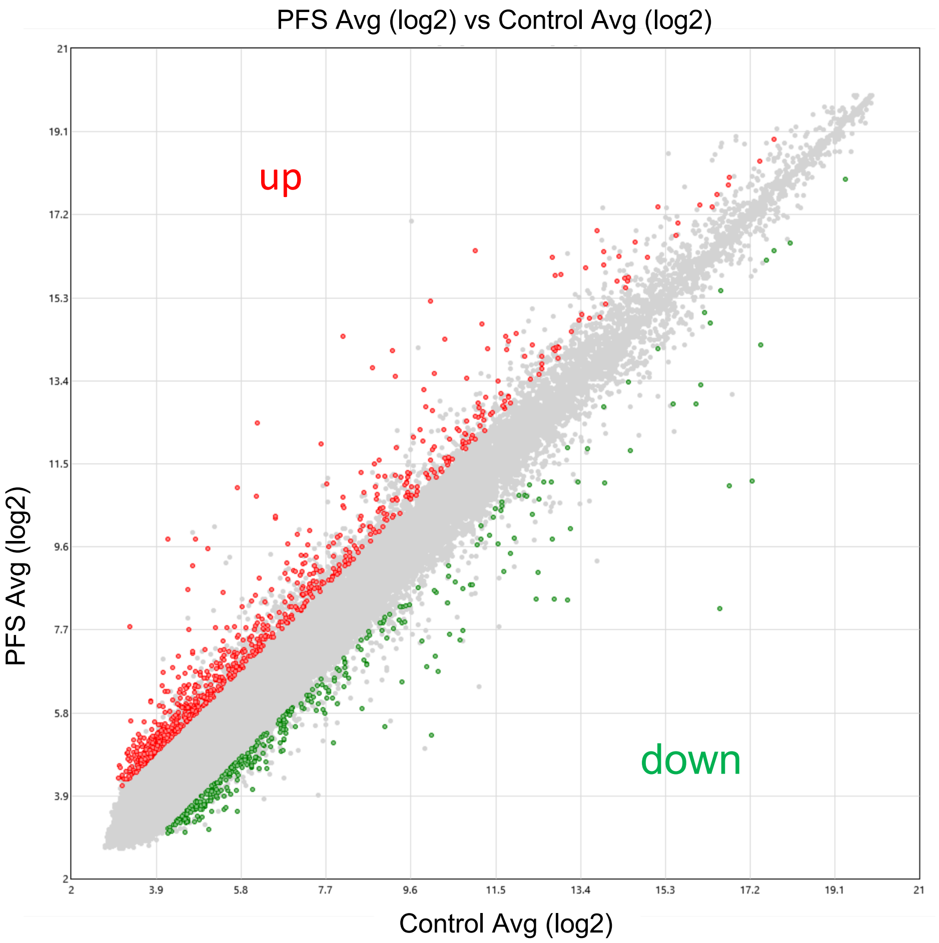

Supplementary Figure S5A. Scatter plot of microarray data showing gene expression changes in PFS-derived fibroblasts vs 4 controls. Scatter plot of normalized probe intensities (log2) for significantly dysregulated genes from the Clariom D microarray analysis of PFS-derived fibroblasts (y axis) vs 4 gender-matched fibroblast controls (x-axis). Each circle denotes a single gene with red circles showing up-regulated genes in PFS (>2 fold; $p<0.05$) and green circles showing down-regulated genes in PFS (>2 fold; $p<0.05$)

Suppl. Fig S5B

B

Pathways	Differentially Expressed Genes
WNT-induced pathways	
PCP pathway	*JUND,MAPK9,**WNT16,WNT5B***
Wnt/β-catenin signalling	*ACVR2A,CDH3,CTNNB1,**DKK1**,LEF1,LRP1,PPP2CB,PPP2R1A,PPP2R2B,PPP2R2C, RARB, **SFRP2**,TCF7,UBD,**WNT16,WNT5B***
Wnt/Ca++ pathway	*NFATC4,PDE6C,PLCB1,PRKCA,REL,**WNT5B***
Wound Healing	
HOTAIR Regulatory Pathway	*CTNNB1,ERBB2,LAMTOR5,LEF1,MMP17,MMP2,PIK3CD,REL,SNAI2, STAT3,TCF7,TWIST2,**WNT16,WNT5B***
Rac Signalling	*BAIAP2,CDC42,CDK5R1,CFL2,ITGA2B,ITGA3,ITGA8,LIMK2,MAP2K2,PAK1,PIK3CD, PIP4K2A, PRKCZ,RAF1,REL,RRAS,SH3RF1,TIAM1*
Regulation of the Epithelial-Mesenchymal Transition Pathway	*CTNNB1,FGF10,FGF17,FGF5,FGFRL1,HMGA2,LEF1,MAP2K2,MAP2K5,MMP2,NOTCH3, PIK3CD,RAF1,REL,RRAS,SMAD3,SNAI1,SNAI2,STAT3, TCF7,TWIST2,**WNT16,WNT5B***
RhoGDI Signalling	*ARHGAP35,ARHGAP4,ARHGAP9,ARHGDIB,ARHGEF7,CDC42,CDH11,CDH18,CDH3, CFL2, ITGA2B,ITGA3,ITGA8,LIMK2,MYL7,PAK1,PIP4K2A,PRKCA,RHOD,RHOV*
Regulation of Actin-based Motility by Rho	*BAIAP2,CDC42,ITGA2B,ITGA3,ITGA8,MYL7,PAK1,PIP4K2A,RHOD,RHOV*
Epithelial Adherens Junction Signalling	*ACVR2A,BAIAP2,CDC42,CTNNB1,FARP2,LEF1,LMO7,MAGI1,MYL7,MYO10,NOTCH3, RRAS,SNAI1,SNAI2,SSX2IP,TCF7,TUBA3C/TUBA3D*
BDNF signalling	
PEDF Signalling	***BDNF**,GDNF,NGF,PIK3CD,RAF1,REL,RRAS,**SERPINF1**/PEDF,TCF7*
Neuropathic Pain Signalling In Dorsal Horn Neurons	***BDNF**,CAMK1D,GRIA1,KCNN4,KCNQ3,PIK3CD,PLCB1,PRKCA,PRKCZ*
Neurotrophin/TRK Signalling	***BDNF**,CDC42,MAP2K2,MAP2K5,MAP3K5,NGF,PIK3CD,RAF1,RPS6KA1,RRAS,SPRY2*
Corticotropin Releasing Hormone Signalling	*ADCY2,**BDNF**,JUND,MAP2K2,MEF2A,PRKCA,PRKCZ,RAF1,SLC39A7*
G-protein signalling	
CREB Signalling in Neurons	***ACKR3**,ADCY2,ADGRA2,ADGRD1,ADGRF4,ADRA1D,ADRA2A,ADRA2C,AVPR2,CCR3, CHRM3,EDNRA,F2R,**GABBR2**,GHRHR,GPER1,GPR63,GPR65,GPRC5B, GRIA1, HRH4, LGR5,MAP2K2,NTSR1,OXTR,P2RY6,PIK3CD,PLCB1,POLR2K,POLR2L,PRKCA,PRKCZ, PTGER2,QRFPR,RAF1,RPS6KA1,RRAS,SSTR1, TAAR2*
GABA Receptor Signalling	*ADCY2,AP1B1,**GABBR2**,GABRA1,KCNN4,KCNQ3,UBD*
G-Protein Coupled Receptor Signalling	*ADCY2,ADRA1D,ADRA2A,ADRA2C,AVPR2,CHRM3,DUSP1,DUSP4,**GABBR2**,GPER1, MAP2K2,PDE11A,PDE3A,PDE6C,PIK3CD,PLCB1,PRKCA,PTGER2,RAF1,REL,RPS6KA1, RRAS,STAT3*

Supplementary Figure S5B. Differentially expressed genes in cells with *FAAH-OUT* microdeletion.
Patient PFS derived fibroblast cell line and 4 gender matched control cell lines were analysed using microarrays. Selected pathways of interest that contain significantly differentially expressed genes (DEGs) are shown above, the full list of DEGs can be seen in **Supplementary Table S5**.

Suppl. Fig S6

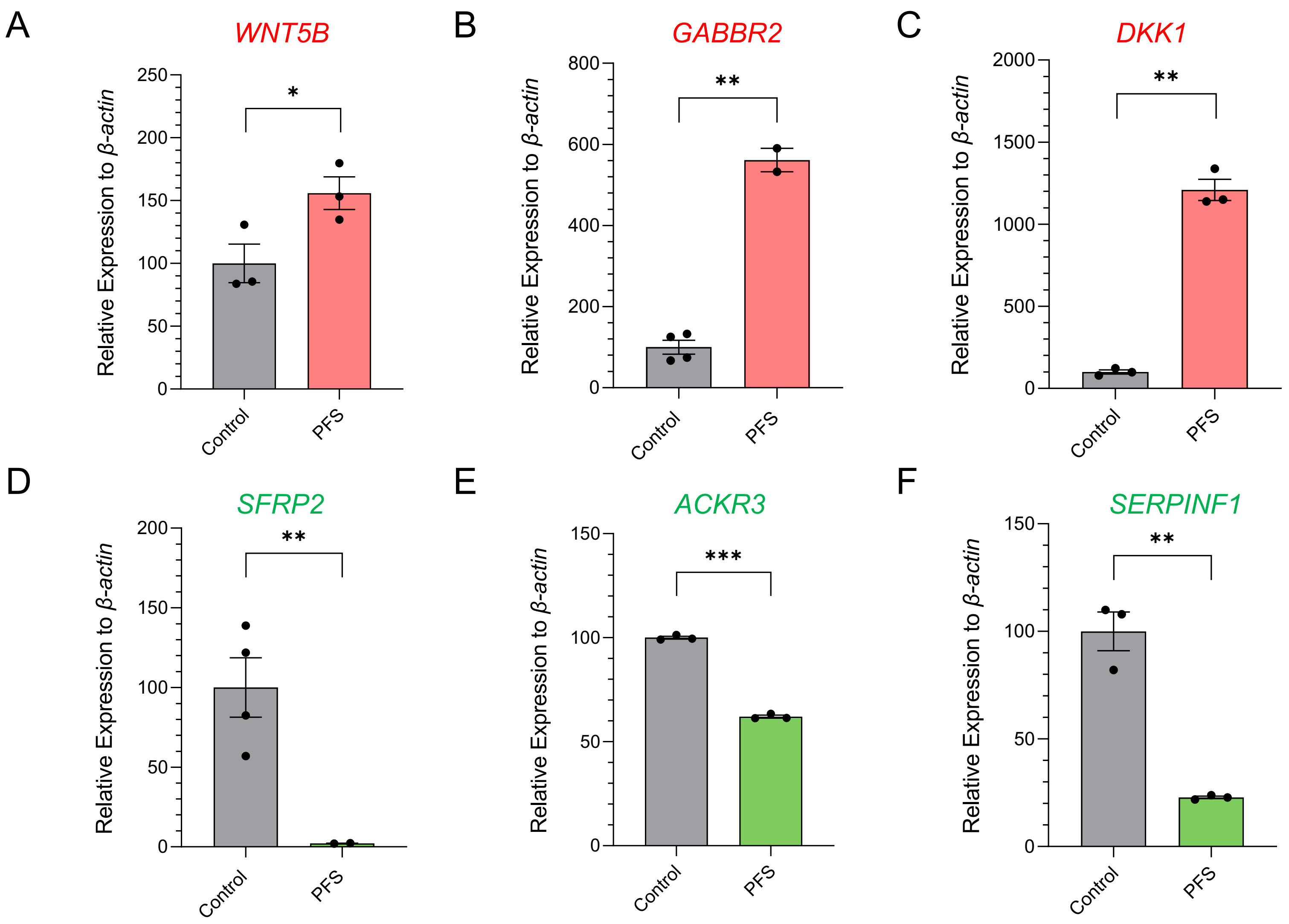

Supplementary Figure S6. Differential expression of key genes in patient fibroblasts confirmed by RT-qPCR.
RT-qPCR analysis of key Differentially Expressed Genes (DEGs) mRNA levels in patient' (PFS) fibroblasts showed significant rise for (**A**) *WNT5B* (1.5-fold); (**B**) *GABBR2* (5.6-fold) and (**C**) *DKK1* (12-fold) expression when compared to gender matched controls. The data also showed significant reduction in expression levels of (**D**) *SFRP2* (47.5-fold); (**E**) *ACKR3* (1.6-fold) and **(F)** *SERPINF1* (4.4-fold) when compared to the control. Data were normalized to beta-actin gene as an endogenous control. The normalized expression value of control subjects was set to 100. Data points are denoted by dots, bars show the ± SEM, and data analysed by Student's t-test, $*P < 0.05$, $**P < 0.01$, $***\ P < 0.001$

A

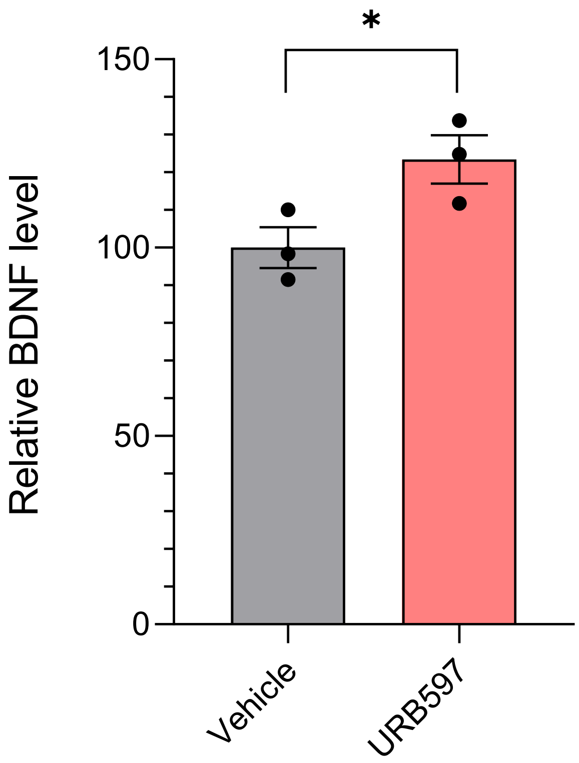

Supplementary Figure S7A. Effects of pharmacological inhibition of FAAH.
BDNF levels rise after FAAH inhibition. Mice treated with FAAH inhibitor URB597 showed significantly higher BDNF (brain-derived neurotrophic factor) levels in the hippocampus (detected by ELISA) compared with vehicle treated mice (n = 3 per group; Data points are denoted by dots, bars show the ± SEM, and data analysed by Student's t-test, $^*P \leq 0.05$).

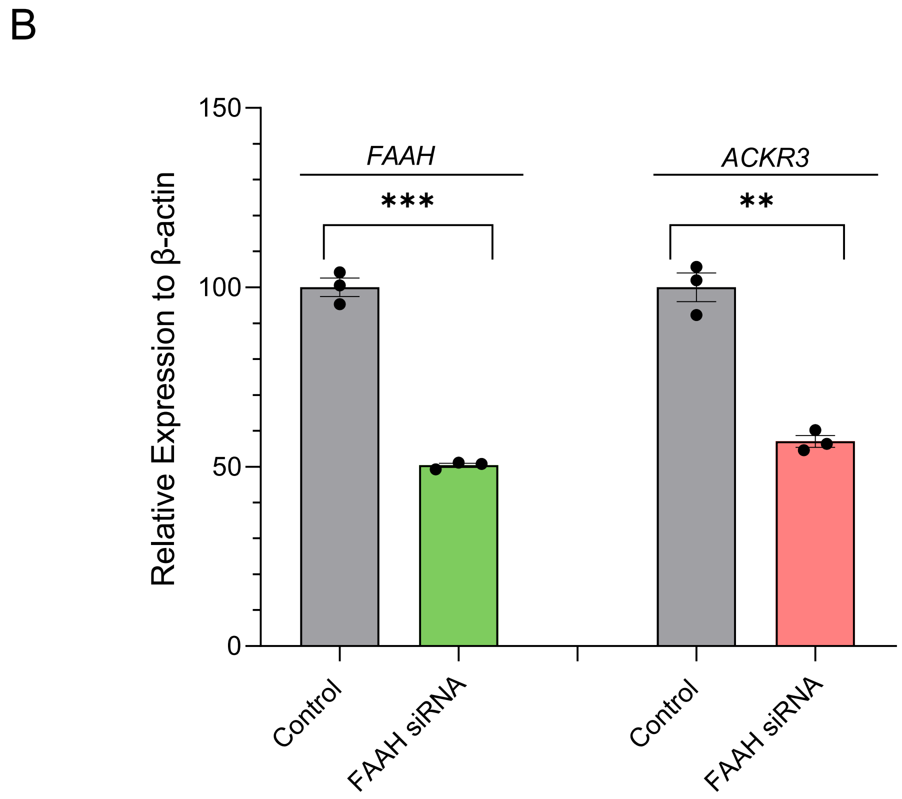

Supplementary Figure S7B. Effects of siRNA knockdown of *FAAH*.

FAAH and *ACKR3* expression in HEK293 cells following *FAAH* siRNA treatment. Expression levels of both genes are significantly reduced after *FAAH* knockdown. Data points are denoted by dots, bars show the ± SEM, and data analysed by Student's t-test $^{**}P \leq 0.01$, $^{***}P \leq 0.001$.

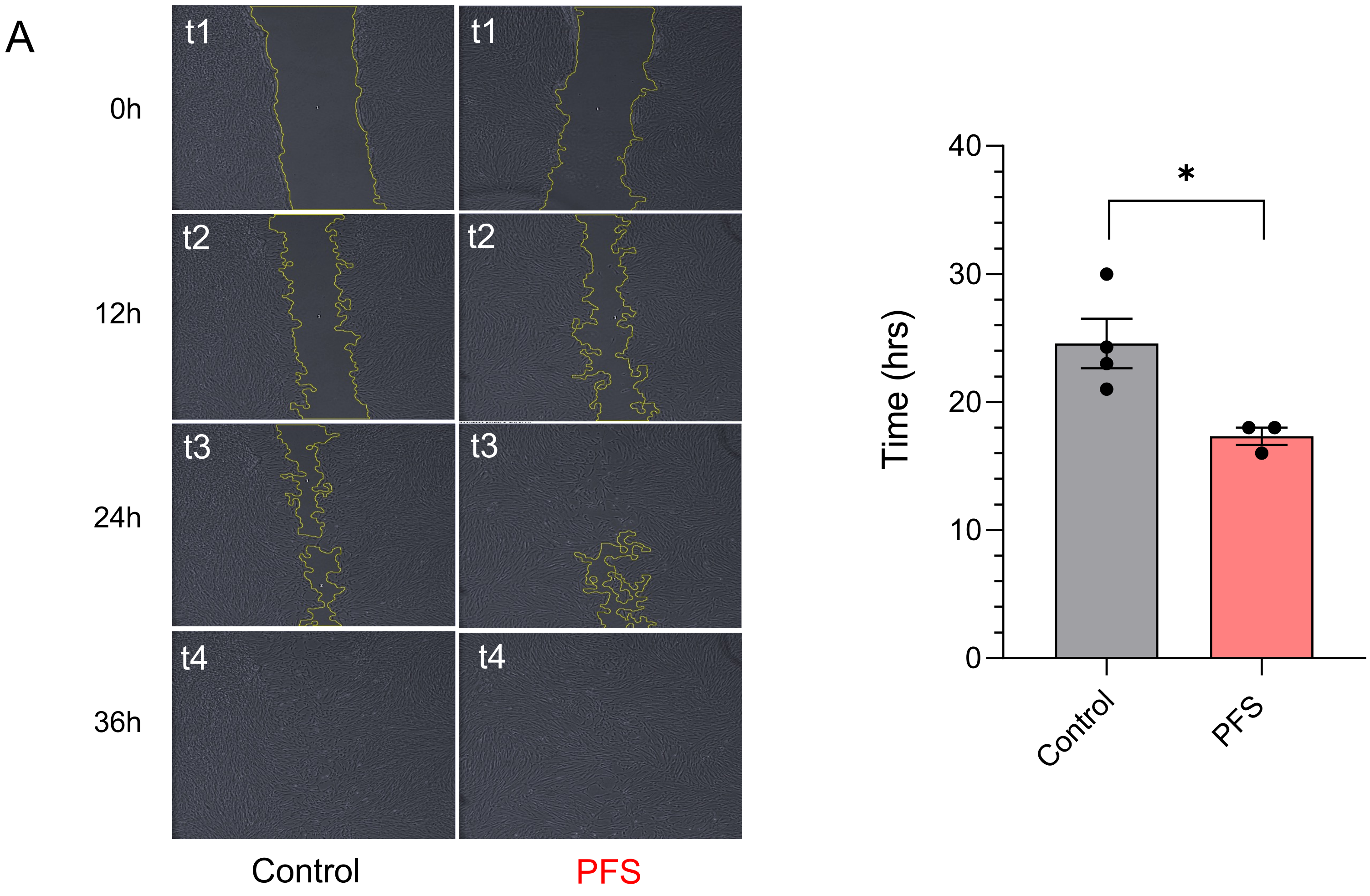

Supplementary Figure S8. The microdeletion in *FAAH-OUT* leads to an acceleration of wound healing.

(A) Wound healing scratch assay was performed using fibroblasts from the PFS patient and a gender matched control who carries the hypomorphic SNP (rs324420) but not the *FAAH-OUT* microdeletion. Images of the recovering monolayer of cells were taken from the start of the experiment (t1=0h) and the following intervals of 12 h for the next 72h (t1, t2, t3, t4 time points correspond to 0, 12, 24 and 36h respectively). **(B)** The images were analysed by Fiji/ImageJ to measure and plot the recovery of the initial gap area (resulting from the scratch) as a function of time. PFS fibroblasts recovered 50% of the scratched gap area in 17h time whereas control fibroblast needed 24.5h to achieve the same, resulting in approximately 30% faster recovery time for PFS fibroblasts. Data points are denoted by dots, bars show the ± SEM, and data analysed by Student's t-test, $*P \leq 0.05$)

Supplementary Table S1: sgRNA sequences

Guide RNA	Sequence	Cas9 type	Location (hg38; Chr1:)
**FOP1**	AAAAGGTGAGGTCACGAGGCC	dSaCas9-KRAB	46,422,643-46,422,663<br>*FAAH-OUT* promoter
**FOC1**	TGTTCCCATGCTTTGGAGTTC	dSaCas9-KRAB	46,424,886-46,424,906<br>FAAH-AMP
***FAAH-OUT* activator**	CTCTCCTGGCGATTGCGATAT	dSaCas9-VPR	46,420,443-46,420,463<br>*FAAH-OUT* promoter
***FAAH* activator**	sc-402470-ACT (Santa Cruz biotechnology)	dCas9-VPR	*FAAH* promoter
**HMa**	CCCAGTGAGTACGATGGCCAG + TTAGTGATATTGTTCCGTGGG	SaCas9	46,418,584-46,418,604 + 46,427,601-46,427,621<br>Flanking patient microdeletion
**HMb**	TCATGGCCTTTCCCCTTCTCA + GTCACTTGCAGTCTGATTAAG	SaCas9	46,418,679-46,418,699 + 46,427,561-46,427,581<br>Flanking patient microdeletion
**FOP2**	CCCAGTGAGTACGATGGCCAG + CCAACCCTCAGGAGTTATGGA	SaCas9	46,418,584-46,418,604 + 46,423,505-46,423,525<br>*FAAH-OUT* promoter + part of exon 1
**FOP3**	TCATGGCCTTTCCCCTTCTCA + GCAACCCCATCCATAACTCCT	SaCas9	46,418,679-46,418,699 + 46,423,496-46,423,516<br>*FAAH-OUT* promoter + part of exon 1
**FOC2**	GCAGCTTTGGCAACTCGGAGT + TGCATGTGTTGGGTTATTGGT	SaCas9	46,424,542-46,424,562 + 46,425,286-46,425,306<br>Flanking FAAH-AMP
**FOC3**	TAGACTAGGGTTCCTGTCTTG + TGTGGGTCTGAATGTCTGGTA	SaCas9	46,424,405-46,424,425 + 46,425,339-46,425,359<br>Flanking FAAH-AMP
**FOC4**	CCCCAGTTTGGAGTCCTCAGT + GGAGGCCTAGGAACCATTACT	SaCas9	Mouse mm10 chr4:115,995,561-115,995,581 + 115,995,214-115,995,234<br>Flanking mouse FAAH-AMP

Supplementary Table S2: Primers used to confirm SaCas9-induced deletions

Guide pairs	Forward primer (5'-3')	Reverse primer (5'-3')
HMa, HMb<br>Primers flanking microdeletion	TTAATGTCTGGAGTGATAACATGAC	ACAACTTCTAATTAGTGTTAATGAC
HMa, HMb<br>Primers internal to microdeletion	TGGATGGTGATTGATGGCCC	GGTCTCGAGGTTGTCACTGG
FOP2,3	GGGACAGAGAGTTGTGCTAATGAAG	CCTCTGTCACATCCTACTCCCAC
FOC2,3	CTGATGGAGGCCATTGGAGGAG	CCAACAGCCCAGACATCCACATG
FOC4	GACAGATGAAAATGGGCTCTGGG	CTCATGAATCGCCCAAGTCATAG

Supplementary Table S3: Primers used for ChIP-qPCR

Region	Forward primer (5'-3')	Reverse primer (5'-3')
FAAH-OUT promoter	ACTTGAAACCCACACGCTCA	AGGCTGGTGTCCGAAGAATG
FAAH promoter	CTGATCCAGTCCGGGTTTTG	CGTACTGCACCATGATCCCTT
FAAH-AMP	TGTCCCCTGGATGGTGATTG	CACCACTGCAACCCATTAGC
ChIP gene desert (control)	AAGAGGCCCTTCCTCTATGC	TGTGATTAATCTCGACTCCAAGA

Supplementary Table S4: Primers used for cellular fractionation

Region	Forward primer (5'-3')	Reverse primer (5'-3')
U6 snRNA	GCTTCGGCAGCACATATACTAAAAT	CGCTTCACGAATTTGCGTGTCAT
ACTB	GGGAAATCGTGCGTGACATTAAGG	CAGGAAGGAAGGCTGGAAGAGTG
