# Supplementary material for: Molecular basis of FAAH-OUT-associated human pain insensitivity
Source: Brain. 2023 May 24;146(9):3851–65. doi: 10.1093/brain/awad098 (PMC10473560; doi:10.1093/brain/awad098)
Supplement: awad098_Supplementary_Data [file awad098_supplementary_data.zip › brain-2022-02087-File012.pdf]

Supplementary Methods

CRISPR/Cas9 plasmids

Plasmids 61591 [1] and 106219 [2] (Addgene) were modified for the gene editing (SaCas9) and transcriptional repression (dSaCas9-KRAB) CRISPR experiments respectively. For plasmid 61591 modification the CMV promoter was replaced with sequence from the *Eef1a1* gene promoter and a shorter polyadenylation sequence. gBlocks gene fragments (Integrated DNA Technologies) were designed to contain a U6 promoter, guide sequence and modified guide scaffold [3] with the design enabling two guide cassettes to be inserted into one SaCas9 plasmid by In-Fusion cloning (Takara). Guide sequences were designed using the online CRISPOR design tool [4]. The sgRNAs chosen were based on a high specificity rank and a low potential off-target score. The 'empty vector' control contained *Eef1a1*-promoter driven SaCas9 but no guide sequences.

SaCas9-IRES-AcGFP1 versions of the plasmids were generated by inserting an IRES-AcGFP1 sequence (Integrated DNA Technologies) after the SaCas9 sequence but before the polyadenylation sequence (at the AflII restriction site) by In-Fusion cloning.

For the transcriptional repression CRISPRi experiments the AgeI-EcoRI fragment from plasmid 106219 containing the dSaCas9-KRAB sequence was used to replace the SaCas9 sequence from plasmid 61591, to give a CMV driven dSaCas9-KRAB. Next, a gBlocks gene fragment (Integrated DNA Technologies) was designed to contain a synthetic poly(A) sequence, U6 promoter, guide sequence and modified guide scaffold [3]. This sequence was cloned into the EcoRI-NotI sites of the modified plasmid 61591 using In-Fusion cloning (Takara). The 'empty vector' control contained CMV-promoter driven dSaCas9-KRAB but no guide sequences. All transformations were performed in Stbl3 *E. coli* strain (Thermo Fisher).

Plasmid 68495 [5] (Addgene) was modified for the transcriptional activation (CRISPRa) experiments. The FseI-EcoRI fragment from plasmid 68495 containing the VP64-p65-Rta

(VPR) sequence was used to replace the KRAB sequence from the CMV driven dSaCas9-KRAB plasmid to give a transcriptional activator plasmid. Next, a gBlocks gene fragment (Integrated DNA Technologies) was designed to contain a synthetic poly(A) sequence, U6 promoter, guide sequence and modified guide scaffold [3]. This sequence was cloned into the EcoRI-NotI sites of the CRISPRa plasmid using In-Fusion cloning (Takara). All guide sequences and their genomic locations are listed in **Supplementary Table S1** and PCR primers used to verify editing are listed in **Supplementary Table S2**.

siRNA assay

In a six-well tissue culture plate, 10^5 HEK293 cells per well were seeded in 2 ml antibiotic-free normal growth media supplemented with FBS. Transfections were carried out using 30 pmols of MISSION esiRNA targeting human *FAAH* (EHU098921; Sigma Aldrich) or a universal negative control (SIC001) with Lipofectamine RNAiMax transfection reagent (ThermoFisher), according to the manufacturer's recommendations. Forty-eight hours after transfection, RNA was isolated and TaqMan real-time PCR was carried out.

Cellular fractionation

Cytoplasmic and nuclear RNA were isolated with the Cytoplasmic & Nuclear RNA Purification Kit (Norgen Biotek; Cat. 21000) following the manufacturer's manual. The effectiveness of cellular separation was controlled with cytoplasmic and nuclear markers *ACTB* and *U6*, respectively (see **Supplementary Table S4**).

Wound healing (scratch) assay

Primary fibroblast cultures derived from patient PFS and a healthy female control were seeded on 6-well plates and allowed to grow until confluent. The control fibroblast cell line, like PFS, was heterozygous for the hypomorphic SNP rs324420 but did not carry the ~8 kb *FAAH-OUT*

microdeletion. Fibroblasts were serum-starved for 24 h, and the bottom of the dish was scraped with a pipette tip (0.2 mL) to create a standardized cell-free area. Cell migration through the space was tracked with time-lapse microscopy in a Nikon BioStation CT (Nikon, Nikon Instruments Europe BV, Netherlands). There, the cells were cultured at 37°C and 5% CO_2, and phase contrast images of wounded areas were automatically recorded every 12 h during the gap closure. The images were analysed by Fiji/ImageJ to computationally measure and plot the gap area as a function of time and then the time it took for the gap to close to half of its original area was calculated.

ELISA

All experiments were performed in accordance with the UK Animals (Scientific Procedures) Act 1986 with prior approval under a Home Office project licence (PPL 70/7382) and following ARRIVE guidelines. Mice were kept on a 12-hr light/dark cycle and provided with food and water ad libitum.

The FAAH inhibitor URB597 (0.3 mg/kg; i.p.) (Cayman Chemical, MI, USA) was freshly dissolved in vehicle containing 5% dimethylsulfoxide (DMSO), 5% Tween-80 and 90% saline. Controls were given the vehicle only. Adult male C57BL/6 mice (Charles River) were intraperitoneally injected with either URB597 (0.3 mg/kg, n = 3) or vehicle (vol:1 ml/kg, n = 3). Twenty-four hours later, mice were euthanized by CO_2 asphyxiation followed by cervical dislocation. The hippocampus was dissected and BDNF content measured using a commercially available sandwich enzyme-linked immunosorbent assay (ELISA) kit (Quantikine ®ELISA-Total BDNF, R&D Systems, Minneapolis, MN, USA) according to the manufacturer's instructions.

Supplementary References

1. Ran FA, Cong L, Yan WX, *et al*. In vivo genome editing using Staphylococcus aureus Cas9. *Nature*. Apr 9 2015;520(7546):186-91. doi:10.1038/nature14299

2. Thakore PI, Kwon JB, Nelson CE, *et al*. RNA-guided transcriptional silencing in vivo with S. aureus CRISPR-Cas9 repressors. *Nat Commun*. Apr 26 2018;9(1):1674. doi:10.1038/s41467-018-04048-4

3. Tabebordbar M, Zhu K, Cheng JKW, *et al*. In vivo gene editing in dystrophic mouse muscle and muscle stem cells. *Science*. Jan 22 2016;351(6271):407-411. doi:10.1126/science.aad5177

4. Haeussler M, Schonig K, Eckert H, *et al*. Evaluation of off-target and on-target scoring algorithms and integration into the guide RNA selection tool CRISPOR. *Genome Biol*. Jul 5 2016;17(1):148. doi:10.1186/s13059-016-1012-2

5. Kiani S, Chavez A, Tuttle M, *et al*. Cas9 gRNA engineering for genome editing, activation and repression. *Nat Methods*. Nov 2015;12(11):1051-4. doi:10.1038/nmeth.3580
