# Supplementary figures and images for: Molecular basis of FAAH-OUT-associated human pain insensitivity
Source: Brain. 2023 May 24;146(9):3851–65. doi: 10.1093/brain/awad098 (PMC10473560; doi:10.1093/brain/awad098)

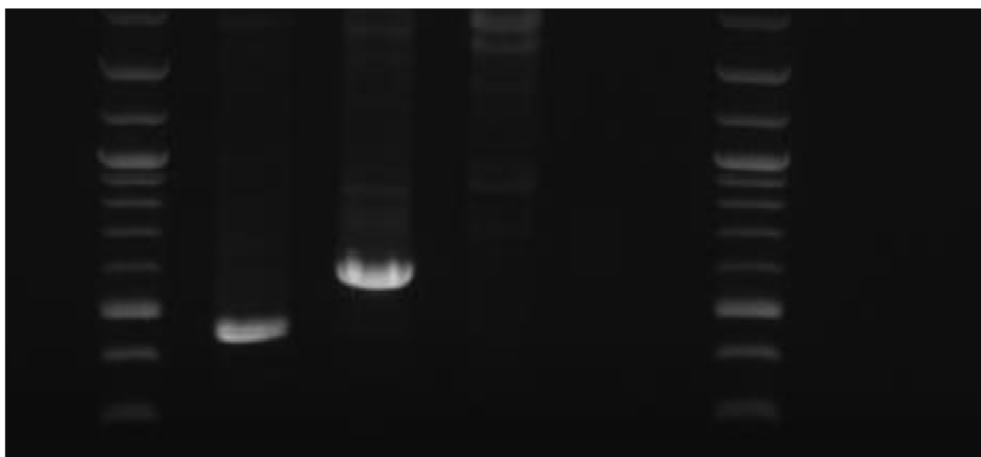

Figure 1B: full-length (uncropped) gel

Supplement: awad098_Supplementary_Data [file awad098_supplementary_data.zip › brain-2022-02087-File013.pdf]
